# Identification of Candidate Adaxial–Abaxial-Related Genes Regulating Petal Expansion During Flower Opening in *Rosa chinensis* “Old Blush”

**DOI:** 10.3389/fpls.2019.01098

**Published:** 2019-09-10

**Authors:** Yu Han, Xue Yong, Jiayao Yu, Tangren Cheng, Jia Wang, Weiru Yang, Huitang Pan, Qixiang Zhang

**Affiliations:** ^1^Beijing Key Laboratory of Ornamental Plants Germplasm Innovation & Molecular Breeding, National Engineering Research Center for Floriculture, Beijing Laboratory of Urban and Rural Ecological Environment, Key Laboratory of Genetics and Breeding in Forest Trees and Ornamental Plants of Ministry of Education, School of Landscape Architecture, Beijing Forestry University, Beijing, China; ^2^Beijing Advanced Innovation Center for Tree Breeding by Molecular Design, Beijing Forestry University, Beijing, China

**Keywords:** rose petal expansion, adaxial–abaxial, auxin, transcription level, *RcREV*

## Abstract

Petal expansion is the main process by which flower opening occurs in roses (*Rosa chinensis*). Although the regulation of leaf expansion has been extensively studied, little is known about the mechanisms controlling petal expansion. The regulation of leaf dorsoventral (adaxial–abaxial) polarity is important for blade expansion and morphogenesis, but the mechanisms involved adaxial–abaxial regulation in petals are unknown. We found that auxin, a key hormonal regulator of leaf adaxial–abaxial patterning, is unevenly distributed in rose petals. The transcriptomes of the adaxial and abaxial petal tissues were sequenced at three developmental stages during flower opening. Genes that were differentially expressed between the two tissues were filtered for those known to be involved in petal expansion and phytohormone biosynthesis, transport, and signaling, revealing potential roles in petal expansion, especially auxin pathway genes. Using a weighted gene coexpression network analysis (WGCNA), we identified two gene modules that may involve in adaxial–abaxial regulation, 21 and five hub genes have been found respectively. The qRT-PCR validation results were consistent with the RNA-seq data. Based on these findings, we propose a simple network of adaxial–abaxial-related genes that regulates petal expansion in *R. chinensis* “Old Blush.” For the first time, we report the adaxial–abaxial transcriptional changes that occur during petal expansion, providing a reference for the study of the regulation of polarity in plant development.

## Introduction

Roses (*Rosa* sp.) are the world’s most popular cut flower and an important horticultural species (Krussmann, 1981; Debener and Linde, 2009). The shape of rose petals determines the shape of the flower, which in turn dictates the ornamental and economic value of each rose cultivar. Flower organ differentiation is largely complete before the rosebud opens; however, the quality of the flower, especially the petal shape and structure (van Doorn and Kamdee, 2014), is established during flower opening. The adaxial (or upper) and abaxial (or lower) epidermal cells of the rose petals are dramatically different from those of the leaves; the petal adaxial epidermis cells have a conical papillate shape, whereas those of the abaxial epidermis are generally larger than those of the adaxial epidermis and have a thicker cuticle (Bergougnoux et al., 2007; Sulborska et al., 2012). Little is known about the mechanisms by which these distinct cell types are formed in rose petals. The concept of adaxial–abaxial (dorsoventral) polarity refers not only to the asymmetry in tissue differentiation relative to the shoot apical meristem but also to the position of the cells relative to the main branch (Soma, 1965; Charlton, 1993; Kaplan, 2001). The establishment of adaxial–abaxial polarity during the early stages of leaf development is an important process regulating leaf expansion, the molecular regulation of which has been examined in many previous studies (Braybrook and Kuhlemeier, 2010; Ishibashi et al., 2013). These findings can be used as references for the study of rose petal dorsiventrality.

As the only known polar transport hormone, auxin plays an important role throughout the plant life cycle, regulating the morphologies of the roots and leaves (Goldsmith, 1977; Blakeslee et al., 2005; Naramoto, 2017). PIN-FORMED1 (PIN1)-mediated auxin transport produces a leaf polarity development signal comprising a lower auxin concentration in the adaxial layer and thereby contributes to the adaxial–abaxial patterning of the leaf (Paponov et al., 2005; Dhonukshe et al., 2007; Qi et al., 2014). The differential gene expression between the adaxial and abaxial surfaces of leaves plays an important role in the patterning of cell differentiation and the ultimate development of the leaf (Nakata and Okada, 2013). At the molecular level, an intricate regulatory network determining adaxial–abaxial polarity has been preliminarily reported in *Arabidopsis thaliana* leaves (Merelo et al., 2017). The class III homeodomain-leucine zipper (HD-ZIPIII) family genes (*REVOLUTA* (*REV*), *PHABULOSA* (*PHB*), *PHAVOLUTA* (*PHV*), and *HOMEOBOX GENE8* (*HB8*)) (Mcconnell and Barton, 1998; Mcconnell et al., 2001; Emery et al., 2003; Prigge et al., 2005; Otsuga et al., 2010), the Myb and LOB domain transcription factors (TFs) *ASYMMETRIC LEAVES1* (*AS1*) and *AS2* (Hidekazu et al., 2002; Lin et al., 2003b; Lin et al., 2003a; Hay et al., 2006; Iwakawa et al., 2010), and the *TAS3*-derived tasiR-ARF (Hunter et al., 2006; Chitwood et al., 2007; Chitwood et al., 2009) have all been identified as adaxial regulators. The abaxial regulators include the *YABBY* family genes (*ABNORMAL FLORAL ORGANS* (*AFO*/*YAB1*), *YAB2*, *YAB3*, and *YAB5*) (Sawa et al., 1999; Siegfried et al., 1999; Stahle et al., 2009; Sarojam et al., 2010), the KANADI family genes (KAN1–3) (Eshed et al., 2001; Kerstetter et al., 2001; Yuval et al., 2004), two *AUXIN RESPONSE FACTOR* genes (*ETT*/*ARF3* and *ARF4*) (Pekker et al., 2005; Chitwood et al., 2009), and miR165/166 small RNAs (Kidner and Martienssen, 2004). The functions of the abovementioned genes in rose petals have not been reported.

Unexpanded rose petals protect the internal floral whorls, and after the flower opens, the petals present different colors and aromas to attract insect pollinators, particularly on the adaxial surface. Flower opening is driven by petal growth, which is largely generated by cell expansion and is associated with the disappearance of the plastids and the enlargement of the vacuoles (Yamada et al., 2009a; Yamada et al., 2009b). Phytohormones, such as auxin and exogenous ethylene, affect petal expansion and flower opening (van Doorn and Kamdee, 2014). *AUXIN RESPONSE FACTOR8* (*ARF8*) regulates petal growth and may be influenced by local auxin levels in *A. thaliana* (Emilie et al., 2011). In postharvest cut flowers of *Rosa hybrida*, ethylene was found to suppress petal cell expansion in a process mediated by *GA-INSENSITIVE1* (*RhGAI1*), which represses the expression of *Cellulose synthase A2* (*RhCesA2*) (Luo et al., 2013). Dehydration can also inhibit petal expansion *via* an ethylene-mediated pathway (Liu et al., 2013).

From flower opening to senescence, the morphology of petals is always changing. Does adaxial–abaxial polarity participate in the regulation of petal expansion in roses? Is this process driven by auxin? Which genes are involved? In this study, we examined the distribution of auxin in the adaxial and abaxial layers of rose (*R. chinensis* “Old Blush”) petals during three stages of floral development, from the flower bud to senescence, using transcriptome sequencing and a weighted gene coexpression network analysis (WGCNA) in addition to the newly released genomic data for this cultivar. Our data will enrich the current understanding of the molecular mechanisms regulating rose petal expansion.

## Materials and Methods

### Plant Growth and Sample Collection

*R. chinensis* “Old Blush” was grown in the glasshouse under a 25°C day/18°C night temperature regime and a 12-h-light/12-h-dark photoperiod. Petals were collected at three typical developmental stages: pink petals in the flower bud (FB_PP), pink petals of the open flower (OF_PP), and senescing petals of the open flower (SF_PP). Freshly harvested petals were immediately separated into upper and lower epidermis samples using a strip of Magic tape (3M, St. Paul, MN), as described previously (Wu et al., 2009). The petal cells were carefully and quickly scraped and collected into a microcentrifuge tube, then frozen in liquid nitrogen. Almost 50 petals were required for one biological replicate to reach 1.0 g of each sample. Three biological replicates were performed for each sample for a total of 18 samples in RNA-seq analysis.

### Petal Phenotype Observation

The adaxial and abaxial petal epidermal surfaces were imaged using a Zeiss Axio Scope A1 (Carl Zeiss, Oberkochen, Germany) following the manufacturer’s recommendations. Five petals were observed in each sample. To observe their internal structures, the FB_PP petals were fixed in paraffin, after which, 8-µm sections were cut from the tip and the center of the petals and dyed with TB (Sigma-Aldrich, St Louis, MO, USA) or PAS (Sigma-Aldrich), according to previously published approaches (O’Brien et al., 1964; O’Brien and McCully, 1981; Day et al., 1995; Tanaka et al., 2004).

### Quantitative Analysis of Indole-3-Acetic Acid Content

The indole-3-acetic acid (IAA) content was quantitatively analyzed following Wang’s previously reported method. For each replicate, 200 mg (fresh weight) petal cells accurately weighted and extracted with 2 ml of cold methanol antioxidant and ^2^H_2_-IAA (internal standard, CDN Isotopes) at −20°C for 24 h. UPLC system (ACQUITY UPLC; Waters) and a triple quadruple tandem mass spectrometer (Quattro Premier XE; Waters) combined to a UPLC-MS/MS system for IAA detecting (Bing et al., 2015). Three biological replicates were performed for each sample, for a total of 18 samples.

### Transcriptome Sequencing and Functional Annotation

An SV Total RNA Isolation Kit (Promega, Madison, WI, USA) was used to extract the total RNA from all samples, following the manufacturer’s instructions. A NanoPhotometer spectrophotometer (Implen, Munich, Germany) and an Agilent Bioanalyzer 2100 system RNA Nano 6000 Assay Kit (Agilent Technologies, Santa Clara, CA, USA) were used to assess the RNA purity and integrity. A total of 18 libraries were constructed and sequenced using an Illumina HiSeq^™^4000 (Illumina, San Diego, CA, USA), which was performed by the Novogene Bioinformatics Institute (Beijing, China). The reference genome and gene model annotation files for *R. chinensis* “Old Blush” were downloaded from its genome website (Raymond et al., 2018) (https://lipm-browsers.toulouse.inra.fr/pub/RchiOBHm-V2/). Bowtie v2.2.3 was used to build the index of the reference genome, and HISAT v2.0.4 with default parameters was used as a mapping tool to assemble transcriptome sequences through alignment with the reference genome (Langmead and Salzberg, 2012; Kim et al., 2015). The raw data were submitted as a BioProject (PRJNA398090) to the NCBI Sequence Read Archive (SRA; http://www.ncbi.nlm.nih.gov/Traces/sra) under accession number SRP115334 and SUB6054346.

All read annotations were retrieved from the “Old Blush” genome annotation RchiOBHm-V2 (https://lipm-browsers.toulouse.inra.fr/pub/RchiOBHm-V2/), including the gene description and the Blast2GO annotation. GO and KEGG ortholog enrichment analyses were performed using the GOseq R package (corrected *P* value < 0.05) and KOBAS v2.0 (corrected *P* value < 0.05; http://www.genome.jp/kegg/), respectively (Kanehisa et al., 2007; Young et al., 2010; Xie et al., 2011). The sequences of *A. thaliana* genes involved in the adaxial–abaxial pathway were downloaded from the TAIR database (http://www.arabidopsis.org/). Gene description and Blast2GO annotations of “Old Blush” genome were used to identify genes involved in the petal expansion, different hormone pathway, and adaxial–abaxial-related genes based on BLAST v2.2.28.

### Analysis of Differentially Expressed Genes

Using the FPKM method (fragments per kilobase of transcript sequence per million base pairs sequenced) (Cole et al., 2010), a differential expression analysis of two groups of genes was performed using DESeq2 (Love et al., 2014). Genes with an adjusted P value < 0.05 were marked as differentially expressed genes (DEGs). Heatmaps of gene expression were produced using pheatmap R package v1.0.10.

### WGCNA

A total of 18 samples in dataset PRJNA398090 and four samples in our previously published dataset SRP092271 (FB_PP1, FB_PP2, OF_PP1, and OF_PP2) (Han et al., 2017) were used to construct a gene coexpression network in the R package WGCNA (Langfelder and Horvath, 2008). Genes that were differentially expressed in at least one comparison (FB_AD vs. FB_AB, OF_AD vs. OF_AB, or SF_AD vs. SF_AB) were subjected to the WGCNA, using limma’s removeBatchEffect to adjust for batch (Ritchie et al., 2015). Three module traits, adaxial (FB_AD, OF_AD, and SF_AD), abaxial (FB_AB, OF_AB, and SF_AB), petal (transcriptome data from the whole petal at stages FB_PP and OF_PP), and eight module traits (FB_AD, FB_AB, OF_AD, OF_AB, SF_AD, SF_AB, FB_PP, and OF_PP) were set. The soft threshold power was calculated using the pickSoftThreshold function. The modules were obtained using the automatic network construction function blockwiseModules with the following settings: power, 6; TOM-type, unsigned; minModuleSize, 30; and mergeCutHeight, 0.25. The eigengenes value was calculated for each module and used to test the associations of each trait; a *P* value < 0.05 was considered significant. The K_ME_, which measures a gene’s connectivity in the specific module, was calculated.

According to the *A. thaliana* reference database in STRING (https://string-db.org/cgi/input.pl) and homologous sequence alignment, the connections between the DEGs in the selected significant module were determined (Szklarczyk et al., 2017). Using CytoHubba from Cytoscape (https://cytoscape.org/) (Chin et al., 2014), the network structure and weighted reconnections between the nodes could be calculated and analyzed using 12 algorithms (including Degree, Edge Percolated Component, Maximum Neighborhood Component, Density of Maximum Neighborhood Component, Maximal Clique Centrality, Bottleneck, EcCentricity, Closeness, Radiality, Betweenness, and Stress), enabling the top 5% most connected genes to be screened as hub genes.

### qRT-PCR Validation

The plant materials used for the qRT-PCR validation included the root, stem, leaf, prickle, stamen, pistil, and ovary, in addition to the adaxial and abaxial epidermal cells of rose petals in the three developmental stages (FB_PP, OF_PP, and SF_PP). Total RNA was extracted using an SV Total RNA Isolation System (Promega), according to the manufacturer’s instructions. The first-strand cDNA was synthesized from 1.0 µg total RNA using a PrimeScript RT Reagent Kit with a gDNA Eraser (Takara Bio, Shiga, Japan). The reactions were carried out on a CFX96^™^ real-time system (Bio-Rad Laboratories, Hercules, CA, USA) using a reaction solution containing 10 µL of SYBR Premix Ex Taq (Takara Bio), 0.4 µL each of the 10-µM forward and reverse transcript-specific primers, 2 µL of cDNA, and 7.2 µL sterile distilled water (dH_2_O). The reaction conditions were as follows: an initial denaturation at 95°C for 30 s, followed by 40 cycles of 95°C for 5 s and 60°C for 30 s, with a final melting-curve stage of 95°C for 15 s, 60°C for 1 min, and 95°C for 15 s. The relative expression levels of each gene were calculated using the 2^−ΔΔCq^ method (Young et al., 2010) and were normalized to the expression of the endogenous reference genes *RcTUB* (Meng et al., 2013) and *RcACTIN* (Han et al., 2017). Each sample was assessed using three technical replicates for each of three biological repeats. The *R*^2^ correlation was calculated to examine the significant differences between the relative expression of the qRT-PCR results and the Log2FoldChange of the RNA-seq data. Origin9 software (OriginLab, Northampton, MA, USA) was used to generate the histograms.

## Results

### Phenotypic Observation of the Abaxial and Adaxial Petal Surfaces From Flower Opening to Senescence

**Figure 1:**
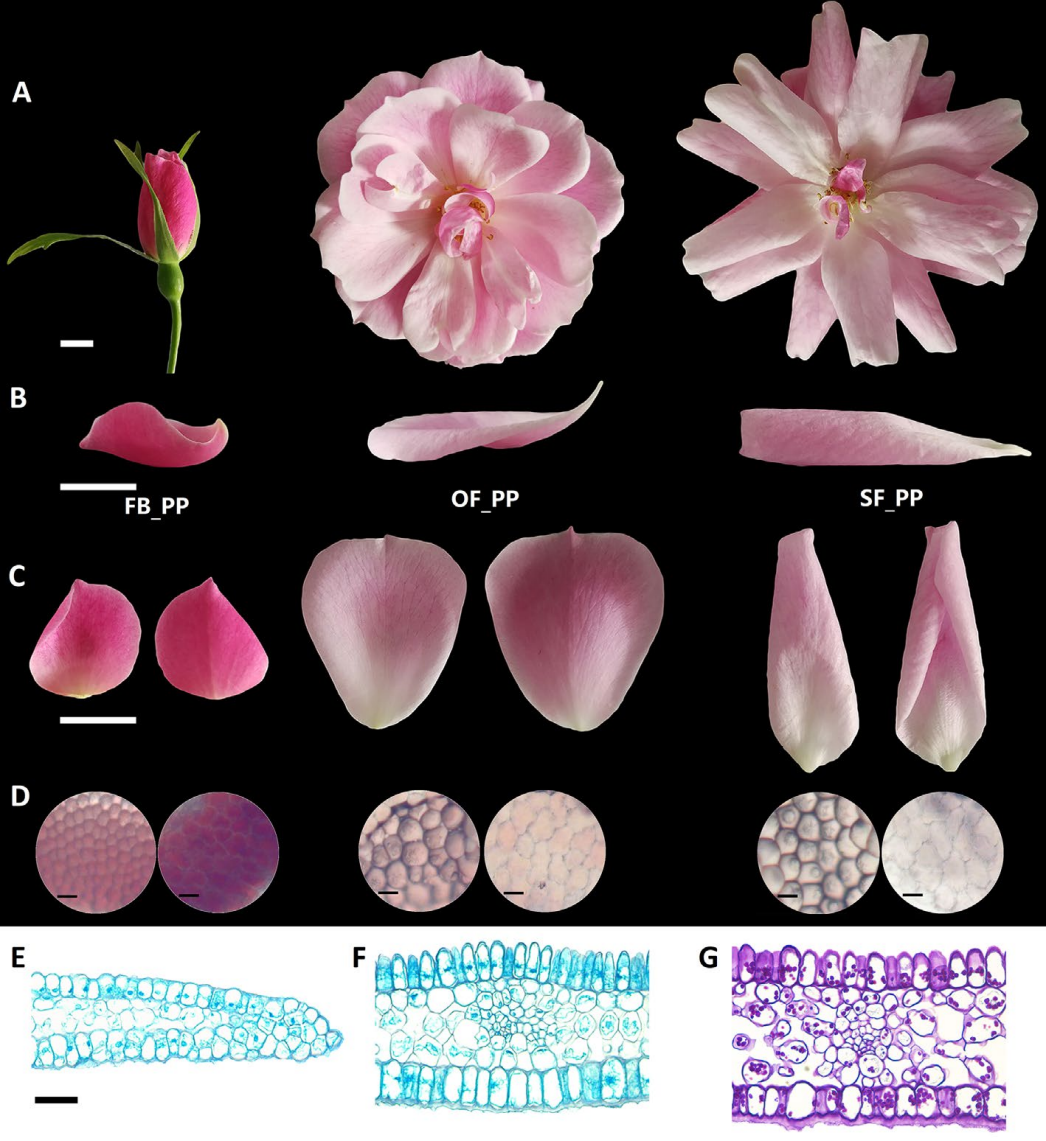
Characterization of the adaxial and abaxial rose petal surfaces. **(A)** The three stages of petals used in this study, from flower opening to senescence: FB_PP, pink petals in the flower bud; OF_PP, pink petals of the open flower; SF_PP, pink petals of the senescencing flower. Scale bar = 5 mm. **(B)** Side view of the petals in the three stages. Scale bar = 1 cm. **(C)** The adaxial (left) and abaxial (right) views of petals in the three stages. Scale bar = 1 cm. **(D)** Microscopy observation of adaxial (left) and abaxial (right) epidermal cells of petals in the three stages. Scale bar = 50 µm. **(E–F)** Microscopy observation of cross sections through the FB_PP petal tip **(E)** and center (Bergougnoux et al., 2007) stained with TB. Scale bar = 100 µm. **(G)** Polysaccharide distribution in a cross section of the center of a FB_PP petal using periodic acid Schiff (PAS) stain. Scale bar = 100 µm.

The petals of “Old Blush” flowers were divided into three typical stages: pink petals in the flower bud before opening (FB_PP), pink petals in the fully open flower (OF_PP), and pink petals in the senescencing flower before they naturally fell off (SF_PP; **Figure 1A**). The side, adaxial, and abaxial views of three petal stages are shown in **Figures 1B, C**. The FB_PP petals resemble a small bowl with a concave adaxial face and wrap around the internal floral whorls. After a dramatic expansion, the petals become larger and flatter to enable the flower to bloom (OF_PP). During senescence, the petals grow a little larger and reflex to the abaxial side in a withering process. A microscopy observation revealed that the adaxial epidermal cells of “Old Blush” petals in all stages are conical papillate, whereas the abaxial epidermal cells are flat with irregular edges (**Figure 1D**). To further observe the similarities and differences in cell morphology between the adaxial and abaxial surfaces, toluidine blue (TB), and periodic acid Schiff (PAS) staining were used. In the tips of the FB_PP petals, the conical papillate shape of the adaxial cells was not obvious (**Figure 1E**). The abaxial surface of the central section of the petals was covered with a layer of wax that could be clearly stained with TB (**Figure 1F**). The polysaccharides of the abaxial and adaxial surfaces were stained with PAS; however, no obvious differences were observed in the polysaccharide distribution of the adaxial and abaxial layers (**Figure 1G**). These results confirm that the petals increase in size from stages FB_PP to SF_PP and have different morphological characteristics on their adaxial and abaxial surfaces.

### Comparative Analysis of DEGs Between the Adaxial and Abaxial Petal Cells

To identify transcriptomic differences between the adaxial and abaxial petal cells in rose flowers throughout development, these tissues were carefully separated and subjected to an RNA-seq analysis. The use of three biological repeats resulted in a total of 18 RNA samples being sequenced; FB_AD and FB_AB, OF_AD and OF_AB, and SF_AD and SF_AB represent the adaxial and abaxial surfaces of petals at stages FB_PP, OF_PP, and SF_PP, respectively (**Supplementary Table S1** and **Supplemental Figure 1**). The genome from a heterozygous diploid of the *R. chinensis* cultivar used in this study, “Old Blush” (RchiOBHm-V2) (Yamaguchi et al., 2012), was used as a reference for the transcriptomic analysis. All clean reads were mapped onto the reference genome data, resulting in genome map rates ranging from 88.07% to 94.90% (**Supplementary Table S1**). The FPKM values were used to estimate the gene expression levels. The DEGs in the adaxial and abaxial samples at the three stages (FB_AD vs. FB_AB, OF_AD vs. OF_AB, and SF_AD vs. SF_AB) were identified in a detailed comparative analysis. A total of 6,020 DEGs were detected in the three comparisons (**Supplementary Table S2**), with 1,882, 692, and 972 DEGs specific to the FB_AD vs. FB_AB, OF_AD vs. OF_AB, and SF_AD versus SF_AB comparisons, respectively (**Supplementary Figure 2**).

A total of 799 DEGs were present in all three comparisons and were further evaluated using Gene Ontology (GO) and Kyoto Encyclopedia of Genes and Genomes (KEGG) Orthology-Based Annotation System (KOBAS) analyses. The three most enriched terms belong to the category “biological process” in the GO analysis were “single-organism process,” “single-organism cellular process,” and “single-organism metabolic process.” The most enriched term in the category “cellular component” was “membrane” (**Supplementary Figure 3A**, **Supplementary Tables S3**and **S4**). The most enriched pathway in the 799 DEGs was “metabolic pathways,” followed by “biosynthesis of secondary metabolites” (**Supplementary Figure 3B**, **Supplementary Table S5**).

### Prediction of Auxin Regulatory Pathways Between Adaxial and Abaxial Petal Cells

**Figure 2:**
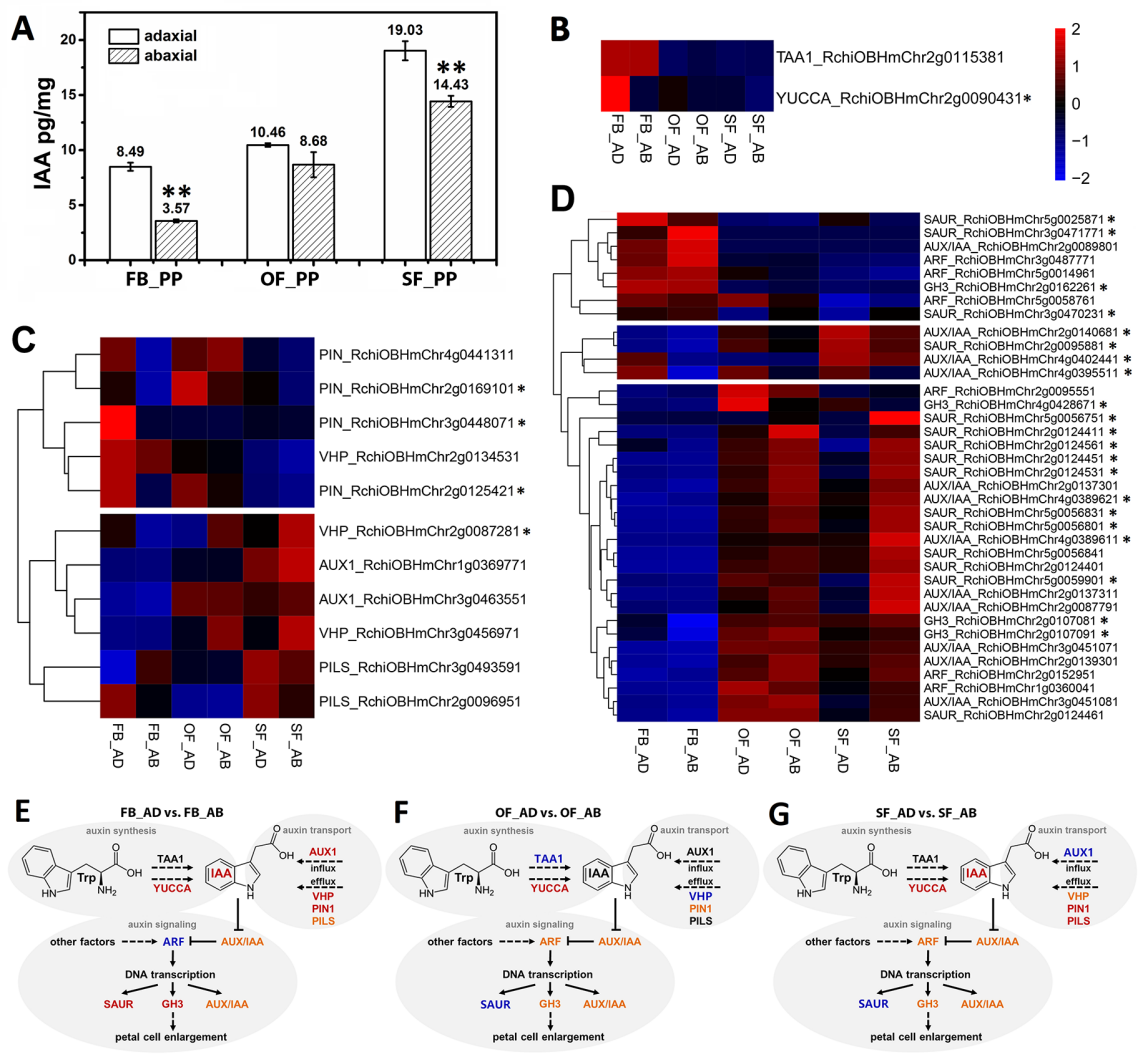
Auxin correlation analysis between the adaxial and abaxial cells of the rose petals. **(A)** Indole-3-acetic acid (IAA) concentrations of the adaxial and abaxial petal cells in the three stages: FB_PP, pink petals in the flower bud stage; OF_PP, pink petals in the open flower stage; SF_PP, pink petals in the senescence flower stage. Three biological replicates for each sample and 0.2 g of petal cells were needed for one replicate. Values are reported as means ± SE (n = 3). Asterisks represent a significant difference to the adaxial surface (*P* < 0.01; Student’s *t*-test). **(B–D)** Heatmaps of the expression of auxin biosynthesis- **(B)**, transport- **(C)**, and signaling transduction-related **(D)** genes between the adaxial and abaxial petal cells in three stages. “FB_AD” and “FB_AB,” “OF_AD” and “OF_AB,” and “SF_AD” and “SF_AB” represent the adaxial and abaxial petal cells in stages FB_PP, OF_PP, and SF_PP, respectively. Red and blue represent up- and downregulated transcripts, respectively. Asterisks indicate the genes with a Log2FoldChange >1 in any comparisons (FB_AD vs. FB_AB, OF_AD vs. OF_AB, and SF_AD vs. SF_AB). All genes are listed in detail in **Supplementary Table S6**. **(E–G)** Summary of transcription-level differences in the auxin pathway between the adaxial vs. abaxial petal cells in the three stages. Black represents no change, red represents an up-regulation, and blue represents a down-regulation; orange groups contain up- and downregulated members. *TAA1*, *TRYPTOPHAN AMINOTRANSFERASE OF ARABIDOPSIS1*; *YUCCA*, *INDOLE-3-PYRUVATE MONOOXYGENASE*; *VHP*, *V-PPASE*; *PIN*, *PIN-FORMED*; *PILS*, *PIN-LIKES*; *AUX1*, *AUXIN INFLUX TRANSPORTER*; *SAUR*, *SMALL AUXIN UPREGULATED RNA*; *AUX/IAA*, *AUXIN-RESPONSIVE IAA*; *ARF*, *AUXIN RESPONSE FACTOR*; *GH3*, *INDOLE-3-ACETIC ACID-AMIDO SYNTHETASE*.

We detected IAA in the adaxial and abaxial petal cells in the three stages (FB_PP, OF_PP, and SF_PP). The IAA content of the adaxial cells was significantly higher than that of the abaxial side in the FB_PP and SF_PP petals (**Figure 2A**). To elucidate the genetic regulation of the IAA pathway in petals, the DEGs were filtered for those previously reported to be involved in auxin biosynthesis, transport, and signaling pathways. A heatmap analysis of the expression (zero-mean normalized FPKM) of DEGs associated with auxin biosynthesis revealed that a *YUCCA* gene (*RchiOBHmChr2g0090431*) had the largest difference in expression between tissues and was most significantly upregulated in FB_AD (**Figure 2B**). Of the auxin transport genes, four auxin efflux carrier *PIN*s (*RchiOBHmChr4g0441311*, *RchiOBHmChr3g0448071*, *RchiOBHmChr2g0125421*, and *RchiOBHmChr2g0169101*) were upregulated in the adaxial cells relative to the abaxial surface at the same stage, especially in FB_PP (**Figure 2C**). Two auxin influx transporter genes, *RchiOBHmChr3g0463551* and *RchiOBHmChr1g0369771* (*AUXIN RESISTANT1*, *AUX1*), were upregulated in the OF_PP and SF_PP stages. Three *ARABIDOPSIS THALIANA V-PPASEs* (*VHP*s) and two *PIN-LIKES* (*PILS*s), which encode putative auxin influx transporters, were differently expressed between the adaxial and abaxial surfaces throughout flower development (**Figure 2C**). We checked the expressions of two auxin biosynthesis genes (*TAA1* and *YUCCA*) and 11 auxin transport genes (*AUX1s*, *PINs*, *PILSs* and *VHPs*) in petal (FB_PP, OF_PP and SF_PP), leaf, stem, root, pistil and ovary by qRT-PCR, one *PIN* (*RchiOBHmChr4g0441311*), and one *PILS* (*RchiOBHmChr3g0493591*) were mainly expressed in petal (**Supplementary Figure 4**).

The DEGs involved in the auxin signaling pathway were divided into two groups in an expression cluster analysis. The first group mainly included genes with significantly higher levels of expression in stages OF_PP and SF_PP than in FB_PP (**Figure 2D**) and was further divided into two subgroups. One subgroup contained three ARFs, three *GRETCHEN HAGEN3* (*GH3*) genes, eight *AUX/IAA*s and eleven *SAUR*s, which were mainly upregulated in OF_AB and SF_AB but had low expression levels in FB_PP stage. The other subgroup contained three *AUX/IAAs* (*RchiOBHmChr2g0140681*, *RchiOBHmChr4g0402441*, and *RchiOBHmChr4g0395511*) and one *SAUR* (*RchiOBHmChr2g0095881*), which upregulated in SF_AD. The other group contained three *SAURs* (*RchiOBHmChr5g0025871*, *RchiOBHmChr3g0471771*, and *RchiOBHmChr3g0470231*), three *ARFs* (*RchiOBHmChr3g0487771*, *RchiOBHmChr5g0014961*, and *RchiOBHmChr5g0058761*), one *AUX/IAA* (*RchiOBHmChr2g008980*) and one *GH3* (*RchiOBHmChr2g0162261*), all of which were highly expressed in the FB_PP stage. All gene information is presented in **Supplementary Table S6**.

In addition to the auxin-associated genes, genes associate with other seven phytohormones biosynthesis and signaling pathways such as abscisic acid (ABA), ethylene (ETH), jasmonic acid (JA), brassinosteroid (BR), gibberellin (GA), cytokinin (CK), and salicylic acid (SA), are displayed in **Supplementary Table S7**, with DEGs in the three comparisons (FB_AD vs. FB_AB, OF_AD vs. OF_AB, and SF_AD vs. SF_AB) presented in **Supplementary Figure 4**. The genes with Log2FoldChange >1 in at least one comparison have been marked with asterisks in the right side (**Figures 2B–D** and **Supplementary Figure 5**). Also includes genes involved in the biosynthesis pathways of ABA, ETH, JA, BR, GA, and auxin signaling.

### Identification of DEGs Involved in Petal Expansion

To elucidate any associations between the regulation of adaxial–abaxial polarity and rose petal expansion, the DEGs in the three comparisons (FB_AD vs. FB_AB, OF_AD vs. OF_AB, and SF_AD vs. SF_AB) were compared against a list of expansion-related genes reported in a previous study (Han et al., 2017). A total of 58 expansion-related DEGs were identified, including six encoding expansins (*EXP*), seven encoding xyloglucan endotransglycosylase/hydrolases (*XET/XTH*), six encoding cellulose synthases (*CES*), 21 encoding pectin esterases (*PE*), four encoding a pectate lyase (*PL*), three encoding polygalacturonases (*PG*), and 11 encoding aquaporins (*AQP*) (**Supplementary Table S8**).

**Figure 3:**
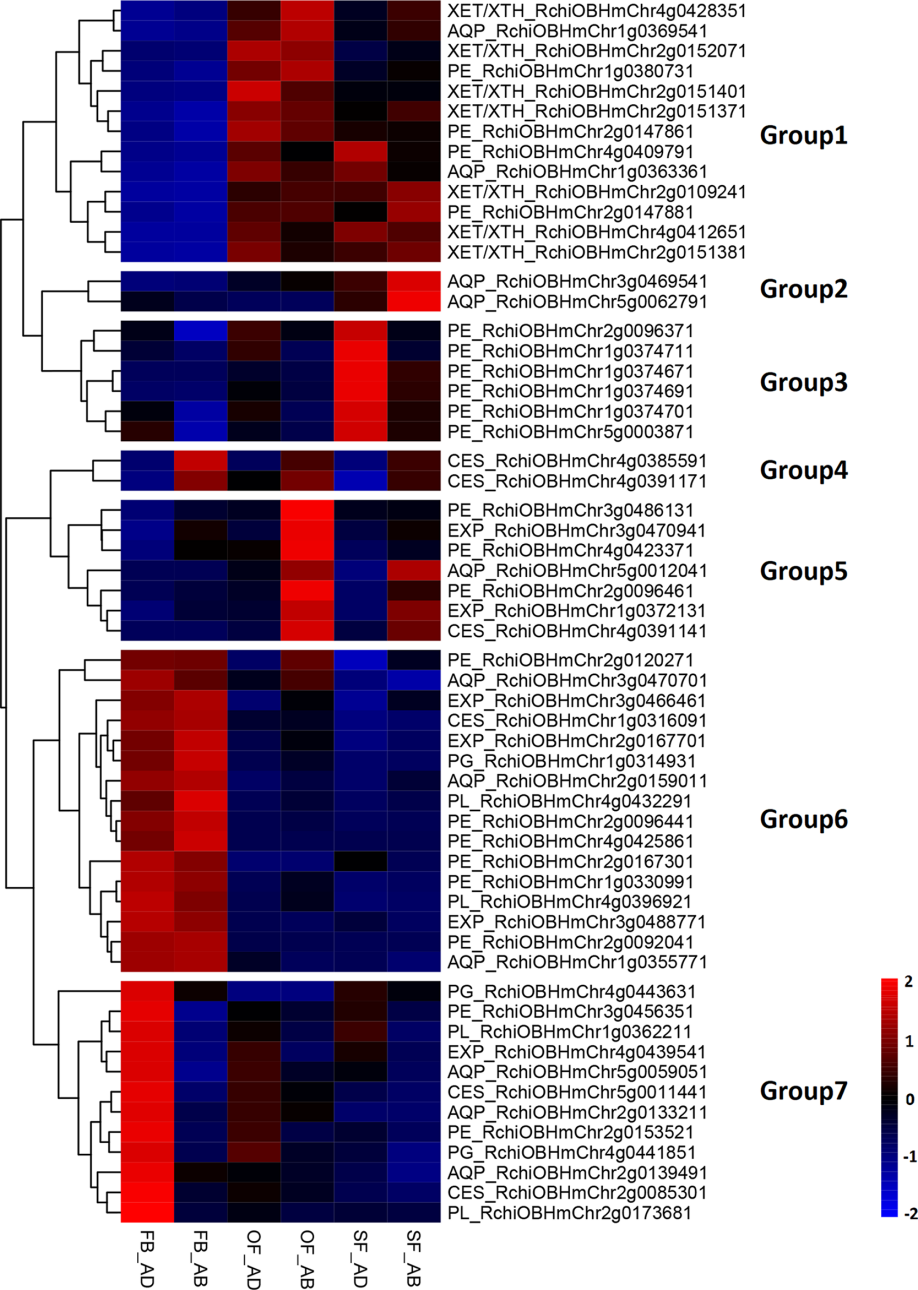
Analysis of petal expansion-related genes differentially expressed between the adaxial and abaxial cells of petals at three developmental stages. Heatmap of petal-expansion-related genes. “FB_AD” and “FB_AB,” “OF_AD” and “OF_AB,” and “SF_AD” and “SF_AB” represent the adaxial and abaxial petal cells in stages FB_PP, OF_PP, and SF_PP, respectively. Red and blue indicated up- and downregulated transcripts, respectively. “Groups 1–7” indicate the nine clustering groups. All genes are listed in **Supplementary Table S8**. *XET/XTH*, *XYLOGLUCAN ENDOTRANSGLUCOSYLASE/HYDROLASE*; *PE*, *PECTIN ESTERASE*; *AQP*, *AQUAPORIN*; *EXP*, *EXPANSIN*; *PL*, *PECTATE LYASE*; *PG*, *POLYGALACTURONASE*; *CES*, *CELLULOSE SYNTHASE*.

Based on their FPKM values, these 58 DEGs were divided into seven groups in a heatmap analysis of their expression patterns (**Figure 3**). Groups 1 contained three types of genes (*XET/XTH*, *AQP*, and *PE*), which were upregulated as the petals expanded during flower opening. In the senescencing petals, two *AQP*s in group 2 were upregulated in the abaxial cells, whereas the six *PE*s in group 3 were upregulated in the adaxial cells. Groups 4 and 5 contained three *CES*s, two *EXP*s, three *PE*s, and one *AQP*, which were upregulated in the abaxial cells. The 10 genes in group 7 comprised genes from six families (*PE*, *AQP*, *EXP*, *PL*, *PG*, and *CES*), which had a low level of expression in the abaxial samples and an expression peak in FB_AD. All genes in groups 6 (*PL*, *PE*, *PG*, *EXP*, *AQP*, and *CES*) were highly expressed in stage FB_PP. In terms of the families of petal expansion-related genes, the *PL*s and *PG*s were mainly upregulated in stage FB_PP, whereas the *XET/XTH*s were upregulated in stages OF_PP and SF_PP in both the adaxial and abaxial cells. All six families of petal expansion-related genes showed differences in expression between the adaxial and abaxial petal cells throughout flower development.

### Elucidation of a Gene Coexpression Module in Adaxial–Abaxial Polarity of Rose Petals

To identify the genes highly associated with adaxial–abaxial polarity during rose petal expansion, we performed a WGCNA using our RNA-seq data. To reduce noise, only genes that were found to be differentially expressed in at least one comparison (FB_AD vs. FB_AB, OF_AD vs. OF_AB, or SF_AD vs. SF_AB) were included. For a more comprehensive analysis, the expression of selected genes in whole petals were also included using the RNA-seq data from our previous study (Han et al., 2017). A total of 6,020 genes were therefore subjected to the WGCNA (**Supplementary Table S9**). The sample clustering and the Pearson correlation coefficient of each sample were analyzed, revealing no outliers in the dataset. The topology of the hierarchical clustering of samples clearly indicated the suitability of the selected genes for a WGCNA (**Supplementary Figures 6A, B**).

**Figure 4:**
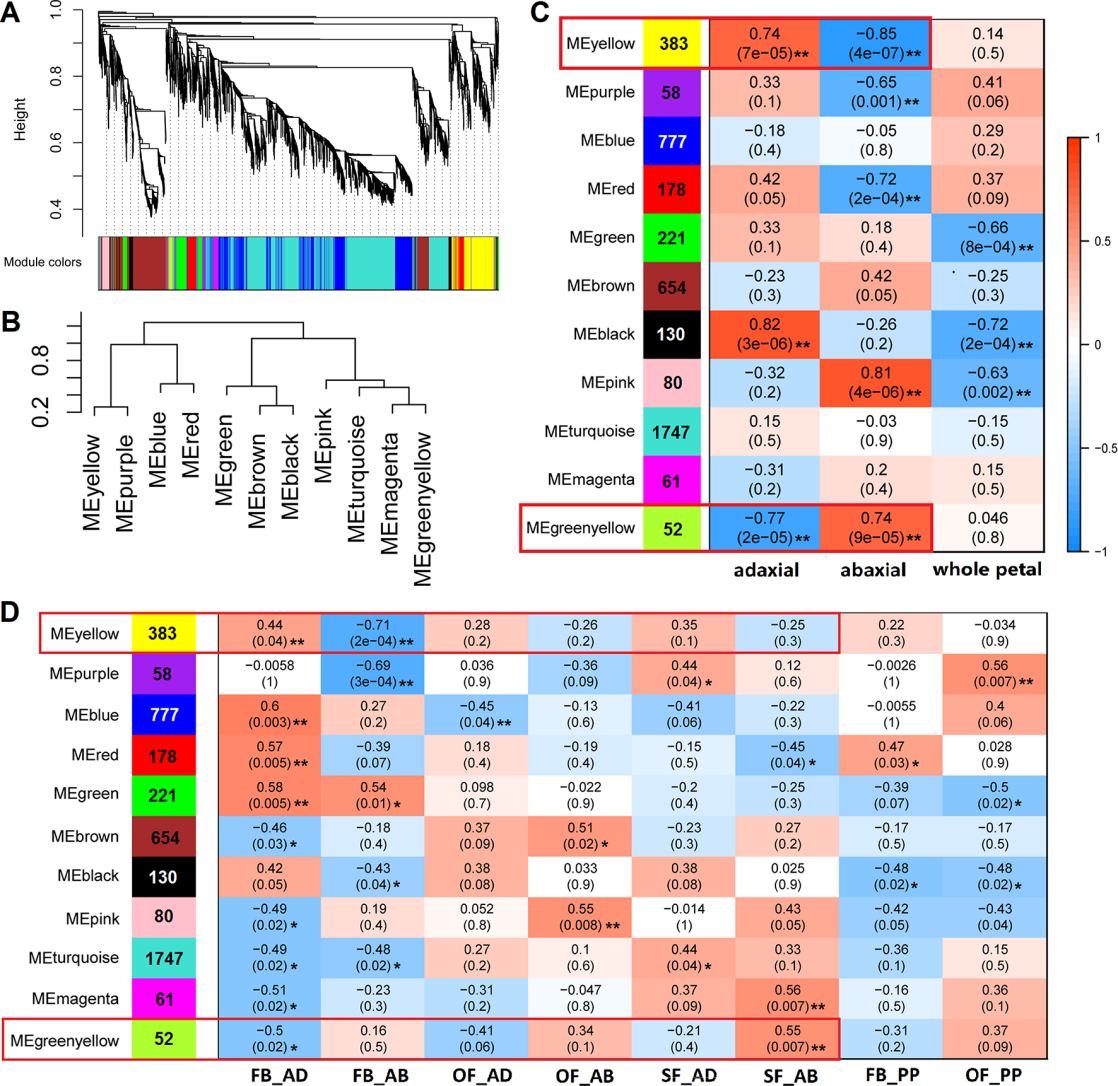
WGCNA of 6,020 DEGs between the adaxial and abaxial petal cells at the three floral development stages. **(A)** Hierarchical cluster tree showing the coexpression modules identified in the WGCNA. One gene is represented by one branch of the tree. Eleven modules make up the major tree branches. **(B)** A heatmap clustering analysis of the 11 modules, labeled with the colors as in **(A)**. Branches of the module group together eigengenes that are positively correlated. **(C)** Module–trait association. Each row corresponds to a module labeled with a color as in **(A)**. The number of genes is marked on the left. Each column corresponds to one trait. “adaxial” and “abaxial” represent all adaxial or abaxial layer samples, respectively, in the three stages, whereas “whole petal” refers to the whole-petal samples in stages FB_PP and OF_PP. The correlation coefficient and significance values are denoted on each module block. A high degree of correlation between the module and traits “adaxial” and “abaxial” is highlighted by the red box. **(D)** Module–trait association of the eight sample groups (FB_AD, FB_AB, OF_AD, OF_AB, SF_AD, SF_AB, FB_PP, and OF_PP). Each row corresponds to a module labeled with a color as in **(A)**. The number of genes is marked on the left. Each column corresponds to one sample group. Significance values at *P < 0.05 and **P < 0.01 are indicated.

The network heatmap plot revealed how the expression of each gene is correlated with every other gene (**Supplementary Figure 6C**). The soft-thresholding power for the analysis of a network topology was set as 6 (**Supplementary Figures 7A, B**). We identified 11 distinct modules (labeled with different colors), as shown in **Figure 4A**. A heatmap clustering analysis revealed the correlation of the 11 modules (**Figure 4B** and **Supplementary Figure 7C**). The gene numbers in these modules ranged from 52 (green-yellow) to 1,747 (turquoise), and the K_ME_ (eigengenes connectivity) value of each gene was calculated (**Figures 4C, D**, **Supplementary Table S10**). We set two types of traits: the sample type (“adaxial,” “abaxial,” or “whole petal”) and the identifier of the eight samples. We found that the yellow module was positively correlated with the adaxial samples and negatively correlated with the abaxial samples, and the green-yellow module was just the opposite (boxed in red, **Figures 4C, D**). These results indicate putatively important roles for the genes in these modules in adaxial–abaxial polarity of rose petal.

### The Selection of Candidate Adaxial–Abaxial-Related Hub Genes in Rose Petals

**Figure 5:**
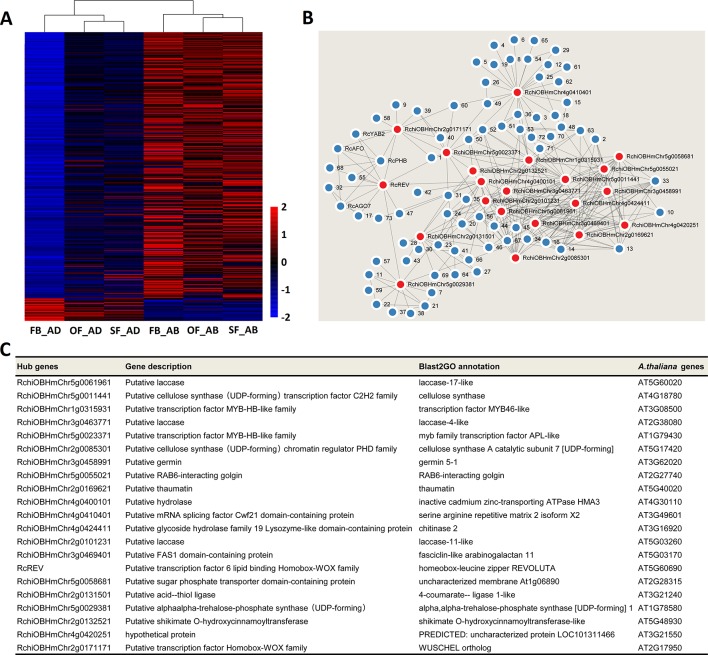
Network analysis of the yellow module of key adaxial–abaxial-related candidate genes. **(A)** Heatmap of the genes in the yellow module. Red and blue represent up- and downregulated genes, respectively. **(B)** Interaction network analysis of the yellow module. Red dots represent hub genes. Blue dots represent genes directly connected to the hub genes. All genes details are listed in **Supplementary Tables S10** and **S12**. **(C)** Functional information on the hub genes in the yellow module.

Heatmaps were constructed to present the expression levels of yellow- and greenyellow-module genes in the abaxial and adaxial cells at each stage of petal development. Most yellow-module genes were upregulated in the abaxial samples and downregulated in the adaxial samples (**Figure 5A**). Most greenyellow-module genes were highly expressed in OF_AB, and SF_AB (**Supplementary Figure 8A**). Based on the gene coexpression data from the WGCNA and the STRING (https://string-db.org/cgi/input.pl) interaction database, we constructed gene networks for hub genes identified in the yellow and green-yellow modules (**Figure 5B** and **Supplementary Figure 8B**) and evaluated their gene connectedness using 12 algorithms (**Supplementary Table S11**). The top 5% of all genes in each module network were selected as hub genes, and their connections to their most close interaction partners were shown in the network (**Figure 5B**, **Supplementary Figure 8B** and **Supplementary Table S12**).

**Figure 6:**
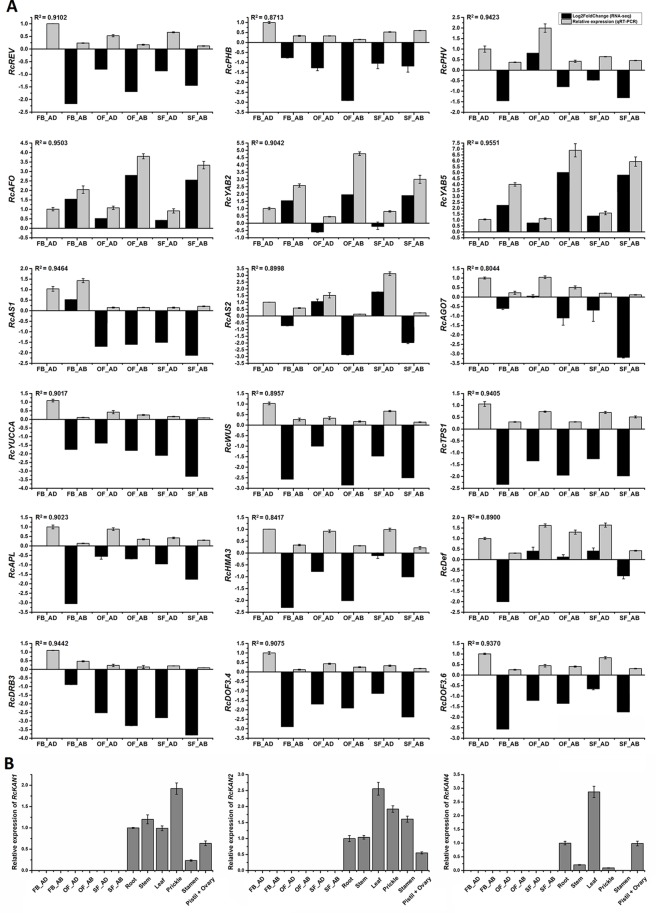
Expression analysis of candidate genes related to rose petal adaxial–abaxial polarity. **(A)** Expression analysis of 18 genes selected from the brown module in six petal samples, using both RNA-seq (Log2FoldChange data of FB_AD vs. FB_AD, FB_AB vs. FB_AD, OF_AD vs. FB_AD, OF_AB vs. FB_AD, SF_AD vs. FB_AD, and SF_AB vs. FB_AD) and qRT-PCR data. Fold changes based on FPKM values (RNA-seq) and expression levels relative to that of the internal controls *RcACTIN* and *RcTUB* (qRT-PCR) are plotted on the same graph. Log2FoldChange (FB_AD vs. FB_AD) = 0. Black columns represent fold changes of the other five samples in comparison with FB_AD. Values of black columns are means adjusted P-value in each comparison. Gray columns represent the relative expression of 18 candidate genes in the six samples. Values of gray columns are means + SD of three biological replicates. R^2^ represents the significance of the correlation between the RNA-seq and qRT-PCR data. The transcript IDs and the primers of each gene are listed in **Supplementary Table S14**. **(B)** qRT-PCR analysis of the *RcKAN1*, *RcKAN2*, and *RcKAN4* transcript levels in six petal samples and other organs (root, stem, leaf, prickle, stamen, pistil, and ovary) of *R. chinensis* “Old Blush.” Values are means ± SD of three biological replicates.

The putative functions of the yellow and green yellow module hub genes and their closest interaction partners were explored to enable the selection of key adaxial–abaxial-related candidate genes (**Figures 5B, C**, **Figures 6B, C**, **Supplementary Table S12**). In the 21 hub genes of the yellow module, four genes were found to encode TFs, including two MYBs, one REVOLUTA, and one WUSCHEL (WUS). The other yellow-module hub genes mainly encoded various enzymes, including laccase, cellulose synthase, and trehalose-6-phosphate synthase (**Figure 5C**). In the five hub genes of the green-yellow module, one gene was found to encode cytochrome P450 84A1-like protein (CYP84A1, *RchiOBHmChr2g0099981*), one encoded receptor kinase belonging to the RLK-Pelle-LRR-XI-1 protein kinase family (*RchiOBHmChr4g0431961*), one encoded an ELO family protein (HOS3-1, *RchiOBHmChr2g0102761*) (**Supplementary Figure 8C**). One strictosidine synthase (SSL3), *RchiOBHmChr5g0049151*, had the highest K_ME_ 0.9648 in the green-yellow module. Detailed information is provided in **Supplementary Table S12**.

A total of 29 genes involved in leaf adaxial–abaxial patterning were previously identified in *A. thaliana*, including seven TF families of leaf polarity determinants and six small RNA pathway components involved in leaf polarity (Ha et al., 2007; Liang et al., 2016). Using the homologous sequence alignment method and the sequences of these 29 *A. thaliana* genes, we identified 39 putative leaf adaxial–abaxial patterning genes in the genome of *R. chinensis* (RchiOBHm-V2), including 27 TFs and 12 small RNA pathway determinants (**Supplementary Table S13**).

### RNA-seq Validation Using qRT-PCR

To assess whether the differentially expressed transcripts could be confirmed using an alternative method, 18 transcripts were selected and analyzed using qRT-PCR (primers listed in **Supplementary Table S14**). The transcripts were chosen on the basis of being a putative homolog of an *A. thaliana* gene with a known function in leaf adaxial–abaxial patterning (*RcREV*, *RcPHB*, *RcPHV*, *RcAFO*, *RcYAB2*, *RcYAB5*, *RcAS1*, *RcAS2*, and *RcAGO7* (*ARGONAUTE7*)) or auxin biosynthesis (*RcYUCCA*), in addition to four hub genes from the yellow module (*RcHMA3* (*HEAVY METAL ATPASE3*), *RcAPL* (*ALTERED PHLOEM DEVELOPMENT*), *RcTPS1* (*TREHALOSE-6-PHOSPHATE SYNTHASE1*), and *RcWUS*) and four genes believed to interact with the core hub gene *RcREV* in the yellow module [*RcDRB3* (*DSRNA-BINDING PROTEIN3*) (32, this number represents the gene location in **Figure 5B**), *RcDef* (*Defensin-like protein*) (47), *RcDOF3.6* (55), and *RcDOF3.4* (68)]. The gene IDs and annotations are listed in **Supplementary Table S12** and **S14**. The R^2^ values between the Log2FoldChange and the relative expression of all genes evaluated using qRT-PCR were all greater than 0.80, demonstrating that the trend of changes revealed using qRT-PCR were consistent with the RNA-seq data (**Figure 6A**). Four of the genes known to be involved in leaf adaxial–abaxial patterning, *RcPHB*, *RcAFO*, *RcYAB2*, and *RcAGO7*, were present in the yellow module network and were found to interact with *RcREV* (**Figure 5B**). KAN is known to regulate organ polarity in *A. thaliana* and is required for the abaxial identity of both the leaves and carpels (Mach, 2014). Three KANs were identified in “Old Blush” (listed in **Supplementary Table S13**) and were named *RcKAN1*, *RcKAN2*, and *RcKAN4* according to the Blast2GO annotation. However, we found that the three *RcKAN*s were not expressed in the adaxial–abaxial petal samples from stages FB_PP to SF_PP. *RcKAN1* was most highly expressed in rose prickles, whereas *RcKAN2* and *RcKAN4* expression was highest in the leaves (**Figure 6B**). Taken together, the detected DEGs form a genetic resource to help understand petal adaxial–abaxial regulation in rose floral opening and senescence.

## Discussion

In *A. thaliana*, the concentration difference of auxin between the adaxial and abaxial surfaces of the leaf primordia is very short-lived. The auxin produced in the adaxial side is transported to the stem cells of the shoot tip, resulting in a low-auxin region on the adaxial side of the primordium and the rapid growth of the abaxial tissues. This asymmetric distribution of auxin results in adaxial–abaxial asymmetry, the expansion of the leaves, and the formation of flat blades (Kitakura et al., 2011;Dong and Huang, 2018). The adaxial–abaxial polarity itself also promotes cell differentiation on both sides of the leaf (Moon and Hake, 2011); upward- or downward-curling leaf phenotypes usually result from a functional mutational in adaxial–abaxial polarity genes and the resulting alteration to the adaxial–abaxial patterning system (Kidner and Timmermans, 2007; Kidner and Timmermans, 2010; Moon and Hake, 2011). However, the regulation of adaxial–abaxial polarity in rose petals seems to be more complicated than that of leaves (Yamada et al., 2009a). The petals are curved to produce different flower patterns, and the flower color and aroma also differ between the adaxial and abaxial surfaces (Bergougnoux et al., 2007). The petal expansion process is accompanied by the further growth of the adaxial and abaxial surface cells. Our results provide a comprehensive analysis of the regulation of adaxial–abaxial polarity in rose petals at a transcriptional level.

The development of rose flowers, from opening to senescence, is accompanied by dynamic growth and morphological changes to the petals (**Figure 1**). The number of DEGs between the adaxial and abaxial petal cells decreased from stages FB_PP to SF_PP (**Supplementary Figure 1**). Auxin was previously suggested to be involved in regulating petal expansion (Emilie et al., 2011), but many of the details remained unclear. Here, we detected that the auxin concentrations of the adaxial and abaxial petal cells were always different (**Figure 2A**) and that the auxin signaling pathway genes were expressed throughout petal expansion (**Figure 2D**). The higher adaxial growth rate may have been caused by these differences in auxin concentration, which may cause the petals to flatten their initial concave shape as they expanded. In stage SF_PP, the adaxial side retained its higher growth rate, causing the senescing petals to reflex towards the abaxial side.

Screening for the DEGs in eight hormone pathways, identifies many auxin pathway genes. IAA, the most abundant auxin in plants, is produced by two main processes: TAA alliinase enzymes (Stepanova et al., 2008; Yi et al., 2008) convert the amino acid tryptophan to indole-3-pyruvate (IPA), which is used by YUCCA-type (YUC) flavin monooxygenase enzymes to produce IAA (**Figures 2E–G**) (Christina et al., 2011). The expression of *YUC* family genes is dependent on adaxial–abaxial patterning. In *A. thaliana*, the blade outgrowth in the *yuc* loss-of-function mutants raises the possibility that auxin participates in the network regulating directed growth activity (Wei et al., 2011). Based on the transcriptome data, we speculated that the auxin biosynthesis genes *TAA1* and *YUC*s; the auxin efflux carriers *PIN*s, *PILS*s, and *VHP*s; and the influx transporter *AUX* are involved in petal expansion. In FB_PP, for example, four *PIN*s, two *VHP*s, and one *PILS* were all upregulated in the adaxial cells, but only one *PILS* was upregulated in the abaxial cells (**Figure 2C**). At the same time, *YUCCA* and *TAA1* were both upregulated in FB_AD (**Figure 2B**), resulting in a higher auxin concentration on the adaxial side that may cause the petal to expand and flatten to promote flower opening. To better represent the transcriptional differences in the auxin pathway genes between the adaxial and abaxial surfaces, three summary sketches of the genes involved in stages FB_PP, OF_PP, and SF_PP are displayed in **Figures 2E–G**. These results demonstrate that the genes involved in auxin transport and signaling are specifically differentially expressed between the abaxial and adaxial petal surfaces throughout flower development, suggesting that they may play an active role in determining and maintaining petal adaxial–abaxial polarity.

To identify the downstream genes of the auxin pathway regulating petal expansion, we analyzed seven types of genes: *PLs*, *PEs*, *PGs*, *AQPs*, *CESs*, *EXPs*, and *XET/XTHs* (**Figure 3**). All of these gene families contained members with both high and low expression in the adaxial and abaxial petal cells. The *EXP*s and *XET/XTH*s have previously been shown to regulate flower opening and petal expansion in roses (Yamada et al., 2009b; Fanwei et al., 2012; Singh et al., 2013). In this study, we found that seven *XET/XTH*s and four *EXP*s involved in petal expansion that were previously uploaded to NCBI by other studies (https://www.ncbi.nlm.nih.gov/, **Supplementary Table S8**) were differentially expressed between the adaxial and abaxial sides. In tomato (*Solanum lycopersicum*) and *A. thaliana*, leaf adaxial–abaxial polarity signals lead to mechanical heterogeneity of the cell wall related to the methyl-esterification of cell wall pectins (Qi et al., 2017). We found that 11 of the DEGs encoded PEs, of which two were significantly upregulated in FB_AD, three were upregulated in OF_AB, and six PEs were upregulated in SF_AD (**Figure 3**), which may be involved in adaxial–abaxial polarity. Our results further refine the transcriptional analysis of the petal expansion genes from the perspective of the adaxial–abaxial.

We set two trait patterns, the sample type (“adaxial”, “abaxial,” or “whole petal”) and the identifier of the eight samples, for analyzing module–trait relationships in WGCNA and added whole petal samples at stages FB_PP and OF_PP as references (**Figures 4C, D**). We identified two gene modules related to adaxial–abaxial. In the yellow module, one of the hub genes was annotated as “homeobox-leucine zipper *REVOLUTA*,” which has a high K_ME_ of 0.9509 and was named *RcREV*. The *A. thaliana* homolog of *RcREV*, *AtREV* (AT5G60690), encodes a TF involved in the regulation of cortical cell development, secondary xylem differentiation in the inflorescence stems, and organ polarity specification of the adaxial–abaxial axis (Emery et al., 2003; Xie et al., 2014). The expression of *RcREV* was consistently higher in the adaxial cells than in the abaxial tissues, consistent with the distribution of the auxin content in the petals (**Figure 6A**). In *A. thaliana*, REV is a major adaxial determinant thought to act antagonistically with the *KAN* genes, which are key regulators of abaxial polarity in the leaves (Yuval et al., 2004; Tengbo et al., 2014). The REV/KAN modules not only regulate auxin biosynthesis, but also affect the activities of the auxin signaling components (such as ARF, SAUR, GH3, and AUX/IAA) and auxin transport components (such as PIN) (Vernoux et al., 2000; Benjamins et al., 2001; Hagen and Guilfoyle, 2002; Cheng et al., 2008; Santner and Watson, 2010; Merelo et al., 2013; Reinhart et al., 2013). Whereas we did not detect the expression of the *KAN* genes in the rose petals at stages FB_PP to SF_PP, they were expressed in other mature organs (**Figure 6B**). The lack of *KAN* gene expression in the expanding petals is notable.

**Figure 7:**
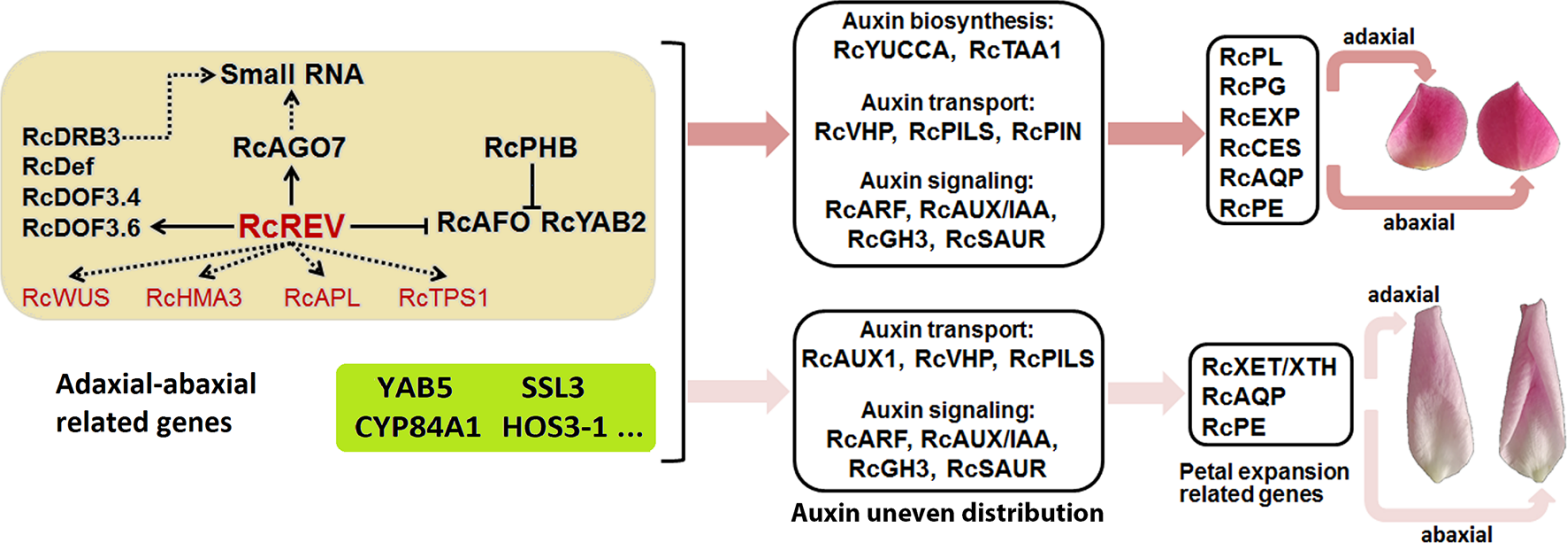
Summary of the adaxial–abaxial polarity regulation of “Old Blush” petal expansion. Hub genes are marked in red. Genes in the light yellow background are candidate adaxial–abaxial-related genes in the yellow module. Genes in the greenyellow background are candidate adaxial–abaxial-related genes in the green-yellow module. Two petals on the top side represent the adaxial (left) and abaxial (right) views of petals in FB_PP stage, another two petals represent the adaxial (left) and abaxial (right) views of petals in SF_PP stage. *YAB5*, *YABBY5*; *SSL3*, *STRICTOSIDINE SYNTHASE-LIKE 3*; *CYP84A1*, *CYTOCHROME P450 84A1-LIKE*; *ELO4*, *ELO HOMOLOG 4*.

In the yellow module, we found there were six xenobiotic-transporting ATPases (*RchiOBHmChr5g0055051*, *RchiOBHmChr4g0427711*, *RchiOBHmChr4g0427721*, *RchiOBHmChr2g0169841*, *RchiOBHmChr2g0091821*, and *RchiOBHmChr4g0427161*) which belong to the ABC transporter B family (ABCB) may associated with auxin. In *A. thaliana*, *ABCB4* encodes an auxin efflux transmembrane transporter (Misuk et al., 2013). In the greenyellow module, most genes was negatively correlated with the adaxial samples and positively correlated with the abaxial samples. An axial regulator, YAB5, belong to this module, and was widely known regulated the abaxial cell fate specification in *A. thaliana* (Yuval et al., 2004). Our results show that the rose *YAB5* (*RchiOBHmChr2g0088171*) was high expressed in the abaxial petal tissues, which may also play important roles in the rose petal adaxial–abaxial polarity regulation. Many of the gene functions of the yellow and greenyellow modules in petal are still unknown. The results of the RNA-seq and the WGCNA, in addition to related clues, such as the regulation of leaf adaxial–abaxial polarity, the auxin pathway, and the function of the petal expansion genes, are summarized in **Figure 7** and are related to the possible biological processes involved.

Our study provides an in-depth analysis of the differing transcriptomes of the abaxial and adaxial petal tissues and proposes a simple network explaining how adaxial–abaxial polarity regulation leads to an uneven distribution of auxin, the expression of downstream petal expansion-related genes, and subsequent petal expansion (**Figure 7**). Building on this network, we will further research the function of various genes and identify those keys to regulating petal expansion. Our research also provides valuable transcriptomic data for petal adaxial–abaxial polarity research, which may enrich our understanding of plant organ polarity development and petal expansion regulation.

## Author Contributions

QZ and YH conceived and designed the study. YH performed most of the experiments, analyzed the data and wrote the manuscript. XY, JY, TC, JW, WY, and HP provided help with the experiments and data analysis.

## Funding

This work was financially supported by the Beijing Natural Science Foundation (6174045), the China Postdoctoral Science Foundation (2017M620650), the National Natural Science Foundation of China (31800594), the Fundamental Research Funds for the Central Universities (2018ZY38 and 2016ZCQ02), and the Special Fund for Beijing Common Construction Project (2016GJ-03).

## Conflict of Interest Statement

The authors declare that the research was conducted in the absence of any commercial or financial relationships that could be construed as a potential conflict of interest.
