# Supplementary material for: Identification of Candidate Adaxial–Abaxial-Related Genes Regulating Petal Expansion During Flower Opening in Rosa chinensis “Old Blush”
Source: Front Plant Sci. 2019 Sep 10;10:1098. doi: 10.3389/fpls.2019.01098 (PMC6747050; doi:10.3389/fpls.2019.01098)
Supplement: Supplementary file 1 [file DataSheet_1.zip › Supplementary Information/Supplementary_Material.docx]

Supplementary Material

# Supplementary Tables

Table S1: Number of RNA-seq reads sequenced and mapped on the *Rosa chinensis* genome.

Table S2: Differential expression information of 6020 DEGs across six samples.

Table S3: Annotation of 799 DEGs in a Venn analysis.

Table S4: GO-enriched terms of 799 DEGs in the Venn analysis.

Table S5: KEGG-enriched terms of 799 DEGs in the Venn analysis.

Table S6: Information and expression of auxin biosynthesis and signaling pathway genes.

Table S7: DEGs information of other seven phytohormones biosynthesis and signaling-pathways.

Table S8: Information and expression of petal expansion-related genes.

Table S9: Read counts of the 6,020 DEGs across 22 samples used for the WGCNA.

Table S10: Gene ID and annotation of all members of the 11 candidate adaxial–abaxial gene modules.

Table S11: Exploration of hub genes in the yellow and greenyellow modules using 12 parameters in CytoHubba.

Table S12: Information on the genes of the yellow- and greenyellow-module networks.

Table S13: Homologs of the *A.thaliana* adaxial–abaxial genes identified in *R. chinensis*.

Table S14: Primer sequences of genes used in the qRT-PCR experiments.

# Supplementary Figures


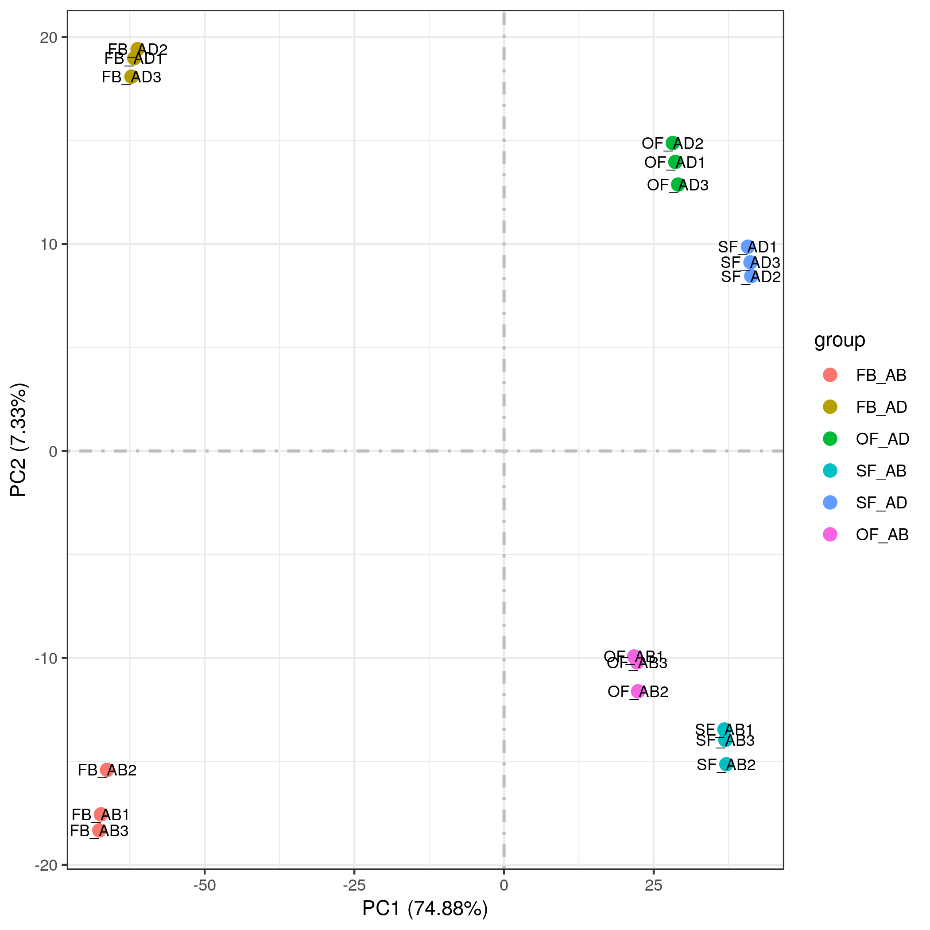


**Supplementary Figure 1.** PCA analysis of the 18 samples. Each group contains three biological replications.


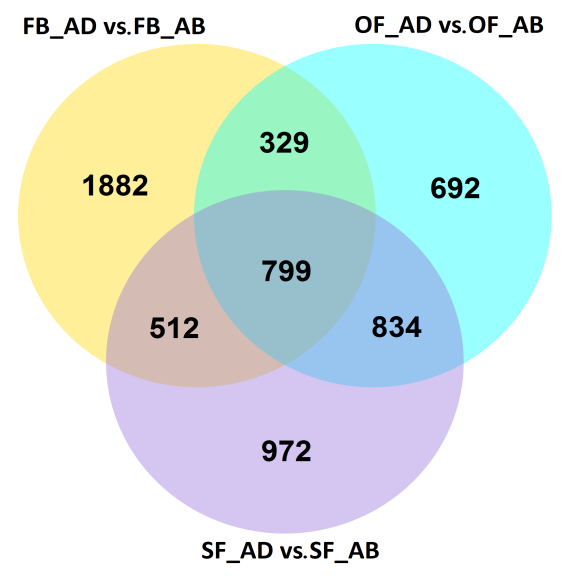


**Supplementary Figure 2.** Venn diagram of the number of differentially expressed genes (padj < 0.05) identified between the abaxial–adaxial comparisons FB_AD vs. FB_AB, OF_AD vs. OF_AB, and SF_AD vs. SF_AB.


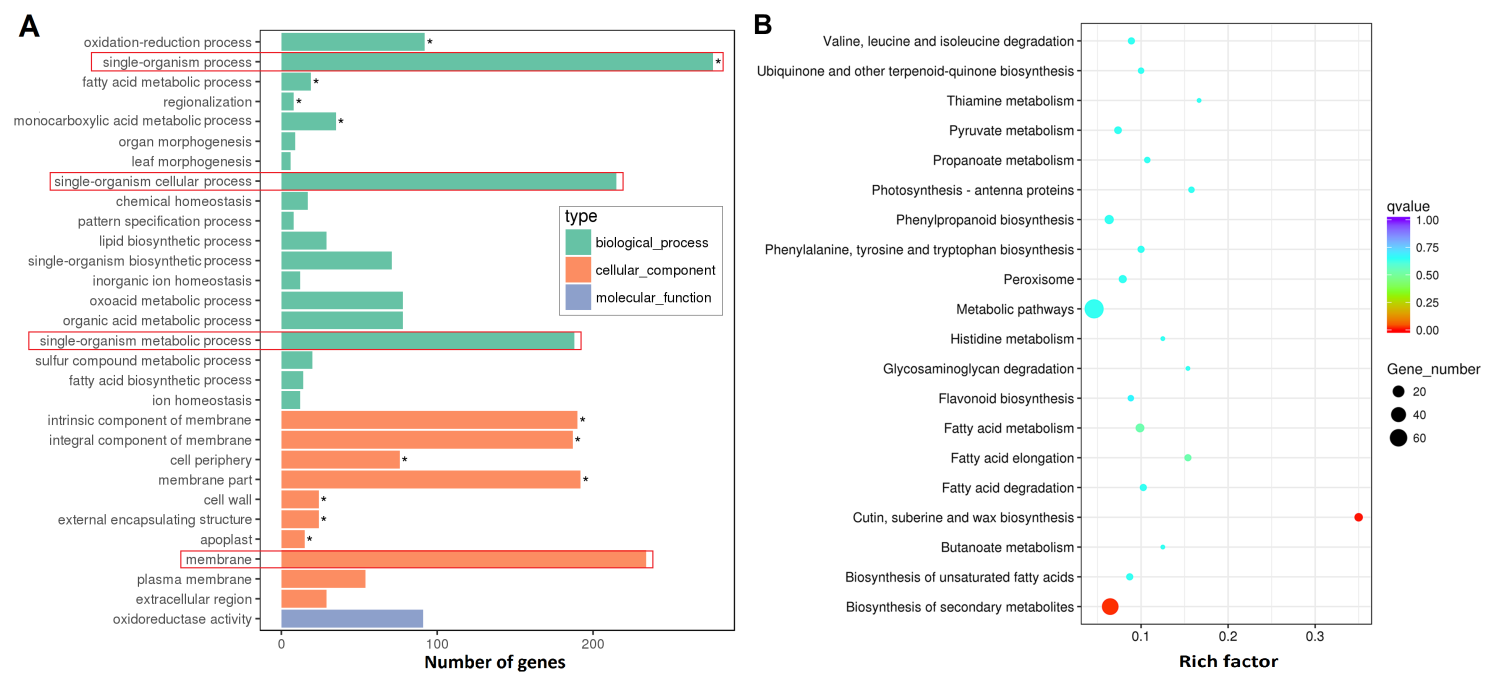


**Supplementary Figure 3.** GO and KEGG analysis of the terms enriched in the 799 DEGs common to the three comparisons (FB_AD vs. FB_AB, OF_AD vs. OF_AB, and SF_AD vs. SF_AB). (A) GO term enrichment analysis of 799 DEGs. The two and one most enriched GO terms belong to biological process and cellular component respectively are highlighted in red boxes. padj < 0.05. (B) KEGG category enrichment analysis of 799 DEGs.

**
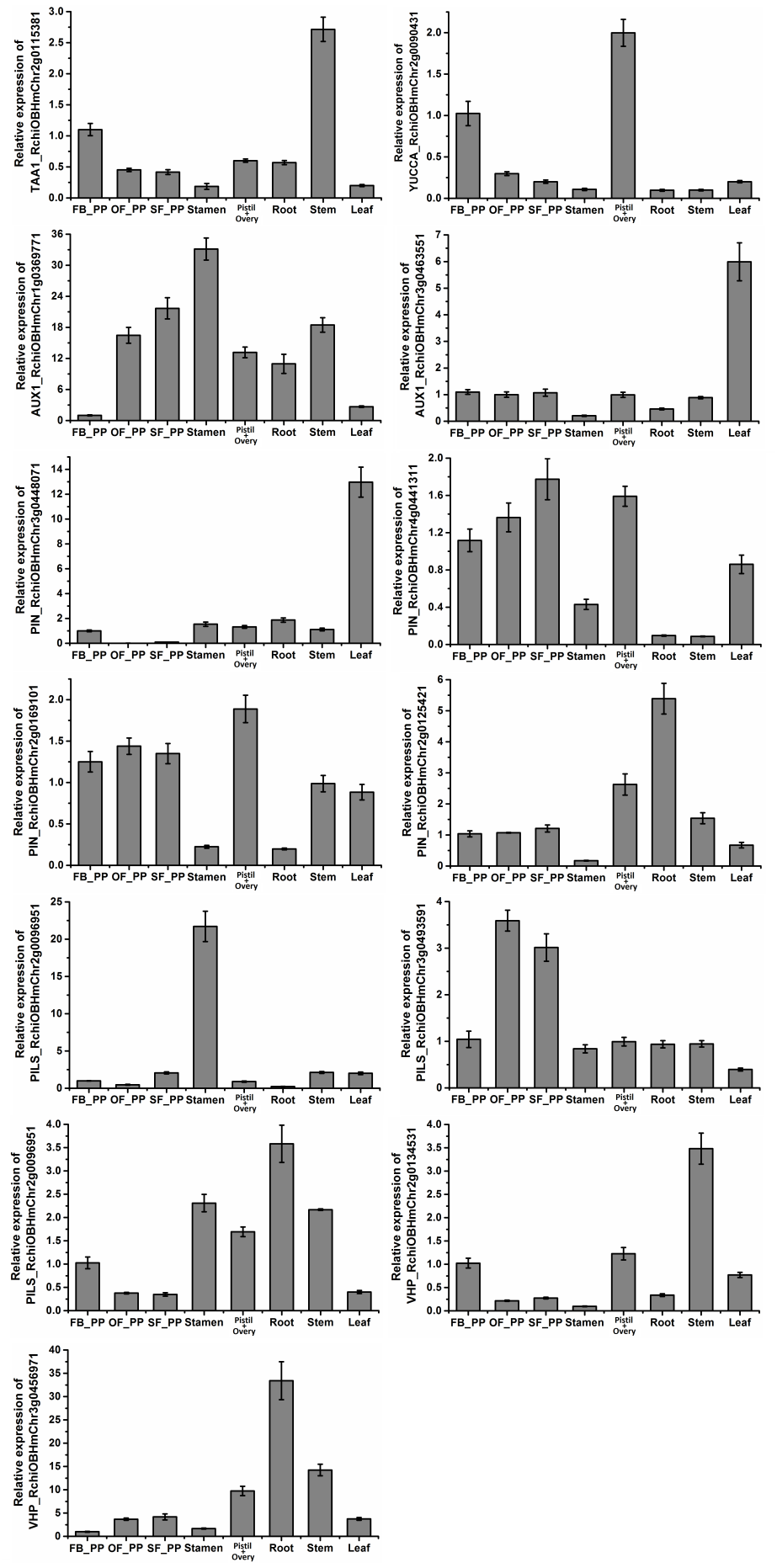
**

**Supplementary Figure 4.** qPCR analysis of the auxin biosynthesis and transport pathway genes in three petal samples (FB_PP, OF_PP and SF_PP) and other organs (root, stem, leaf, stamen, pistil, and ovary) of *R. chinensis* ‘Old Blush’. Values are means + SD of three biological replicates.


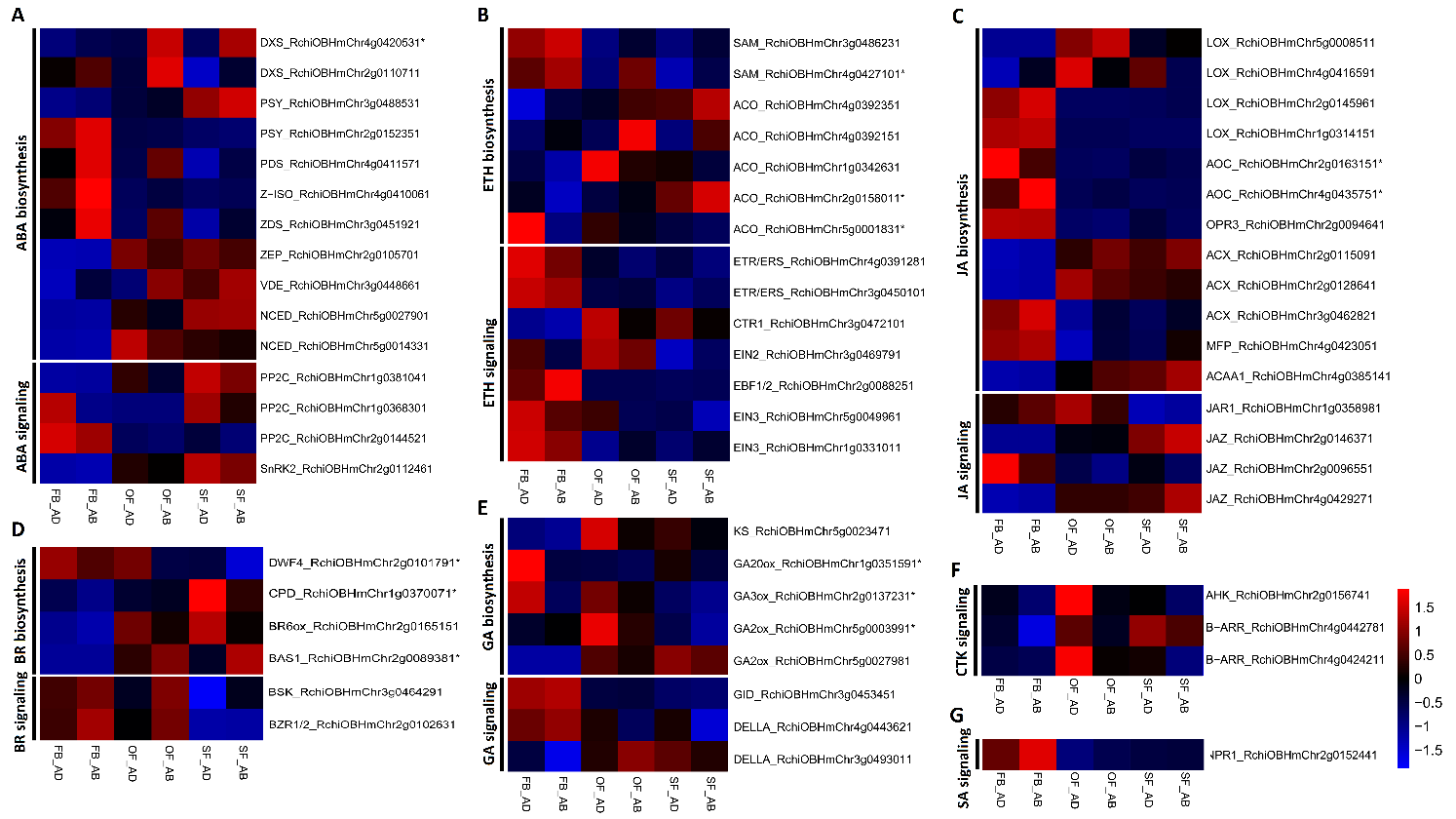


**Supplementary Figure 5.** Heat maps of DEGs involved in the biosynthesis and signaling pathways of (A) abscisic acid, (B) ethylene, (C) jasmonic acid, (D) brassinosteroid, (E) gibberellic acid, (F) cytokinin, and (G) salicylic acid.“FB_AD” and “FB_AB,” “OF_AD” and “OF_AB,” and “SF_AD” and “SF_AB” represent the adaxial and abaxial petal cells in stages FB_PP, OF_PP, and SF_PP, respectively. Red and blue represent up- and downregulated transcripts, respectively. Asterisk indicate the genes with a Log2FoldChange >1 in any comparisons (FB_AD vs. FB_AB, OF_AD vs. OF_AB, and SF_AD vs. SF_AB). All genes are listed in detail in Supplementary Table S7.


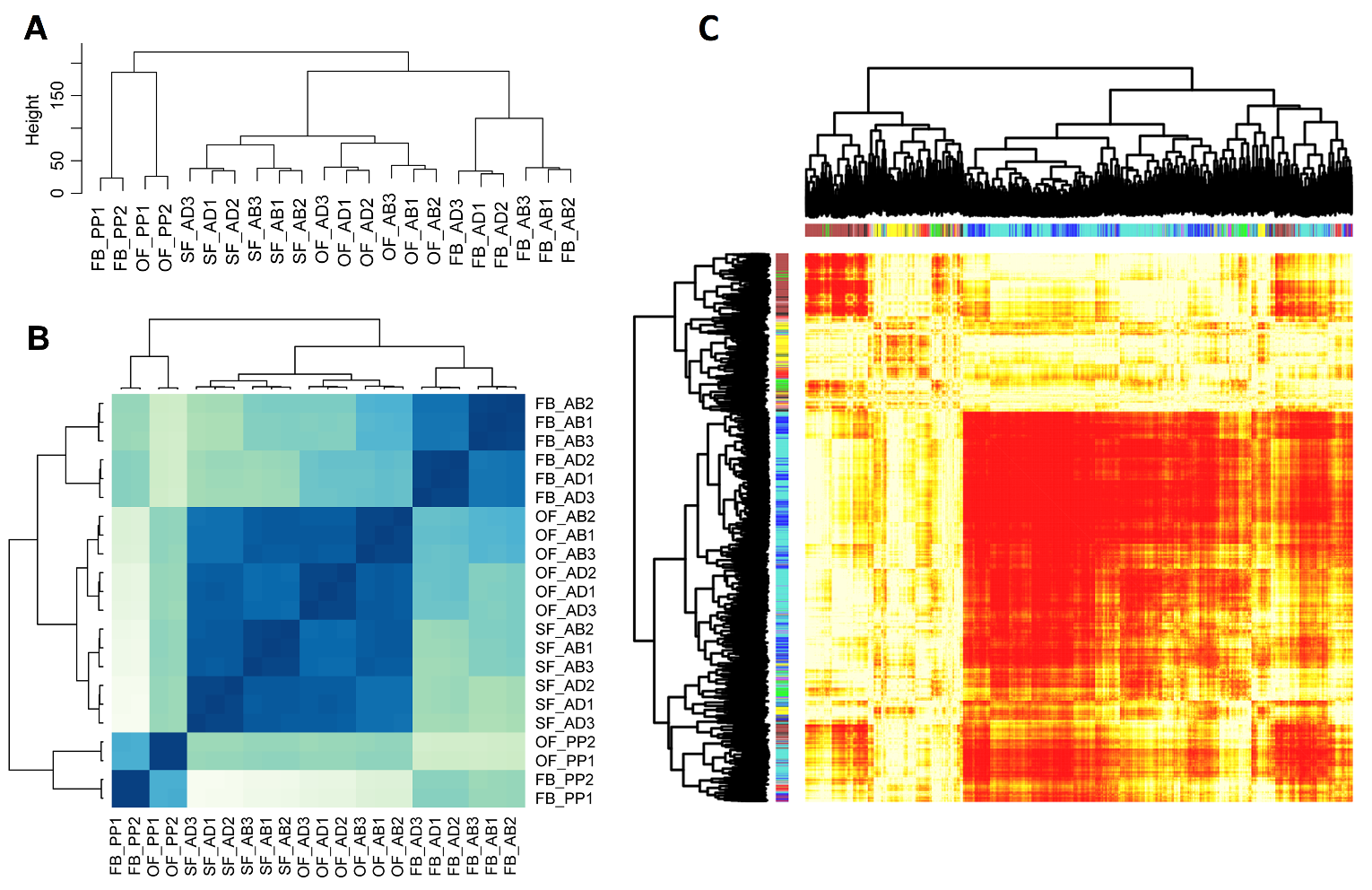


**Supplementary Figure 6.** Cluster analysis of the 22 samples and all genes analyzed in the WGCNA. (A) A 22-sample clustering analysis to detect outliers. (B) Pearson correlation analysis of the 22 samples. (C) Network heatmap plot analysis of all DEGs used in WGCNA.


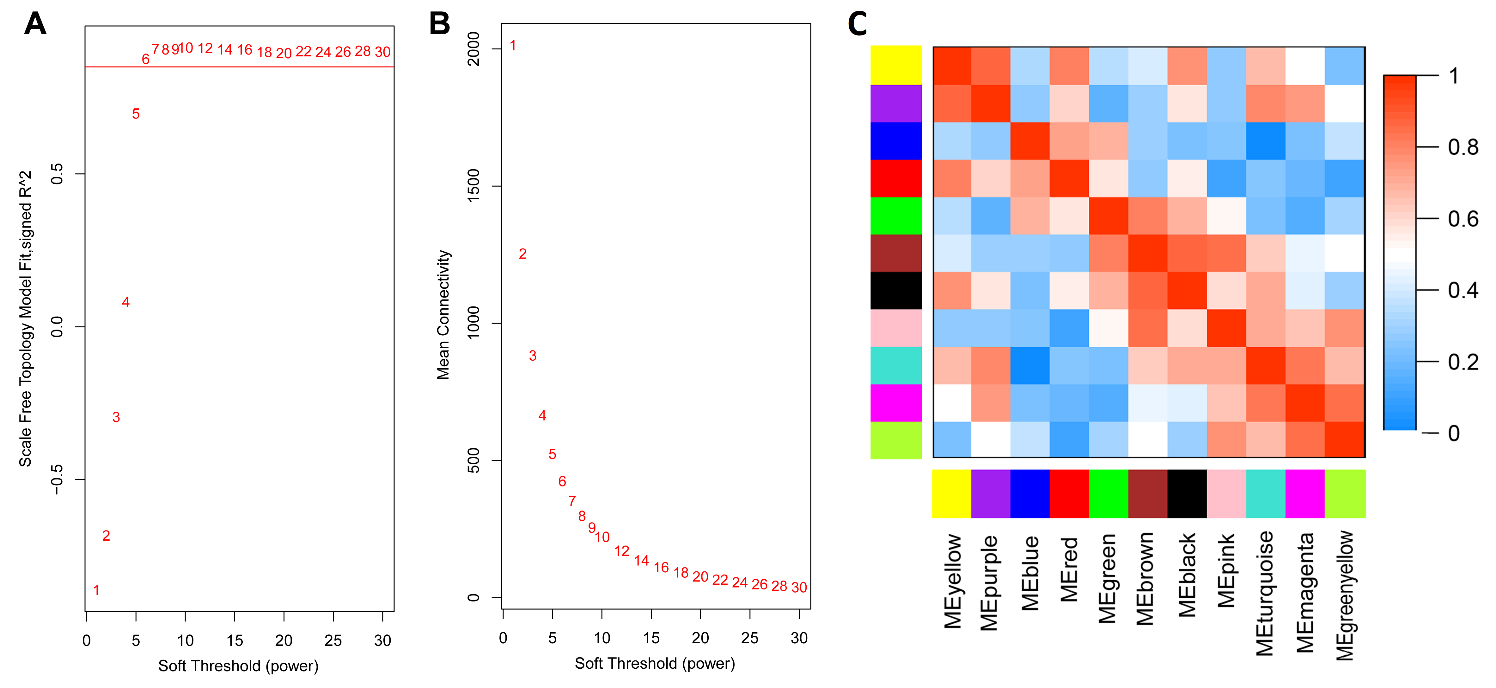


**Supplementary Figure 7.** Soft threshold screening and analysis of the correlation between modules. (A) Soft threshold (power) screening. R^2^ = 0.85. (B) Analysis of the soft threshold and mean connectivity. (C) Heatmap plot of the adjacencies in the eigengene network. Red represents high adjacency; blue represents low adjacency.


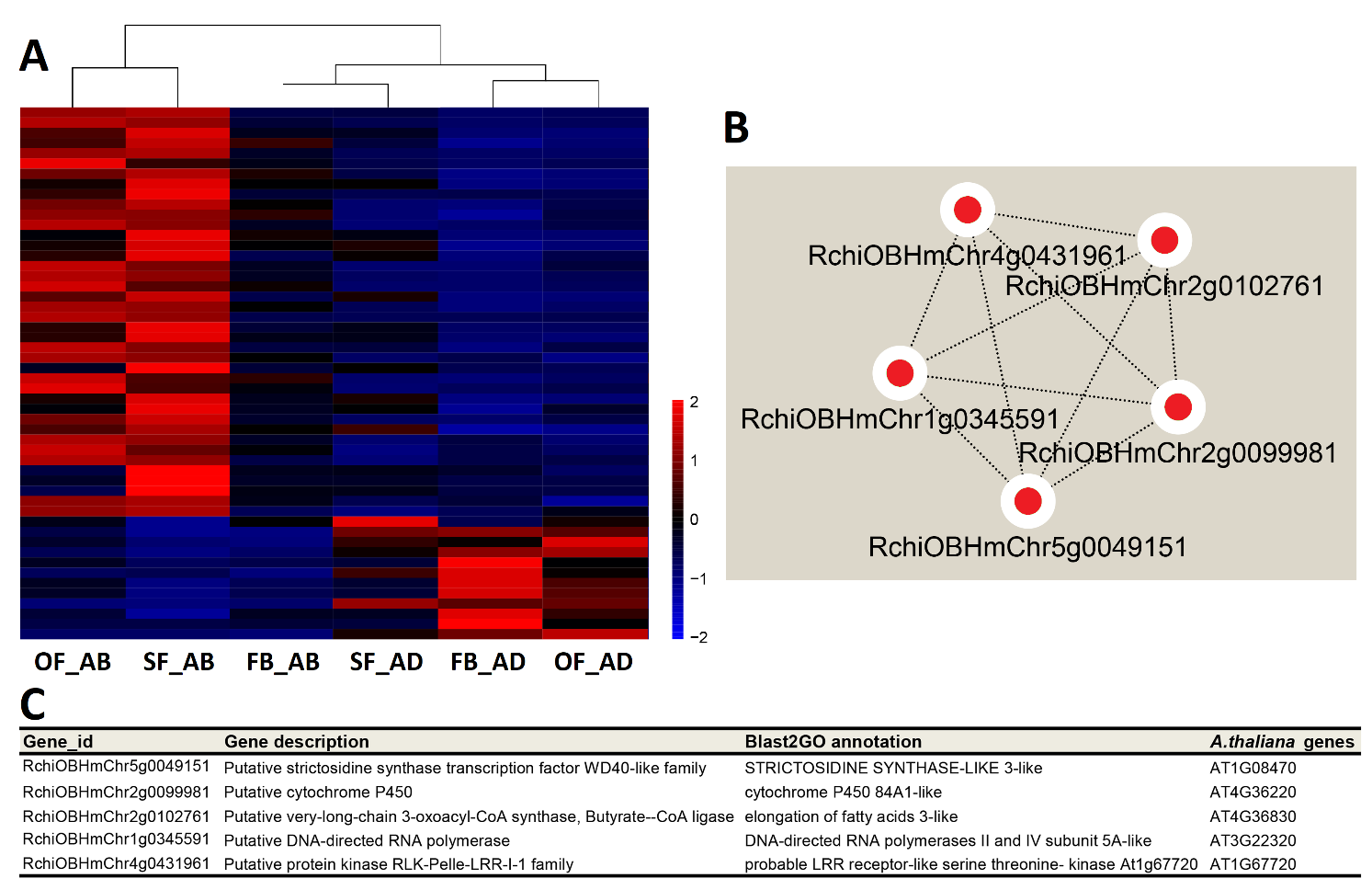


**Supplementary Figure 8.** Network analysis of the greenyellow module of key adaxial–abaxial candidate genes. (A) Heatmap of the genes in the greenyellow module. Red and blue represent up- and downregulated genes, respectively. (B) Interaction network analysis of the hub genes in greenyellow module. Red dots represent hub genes. (C) Functional information on the hub genes of the greenyellow module.
