# Supplementary figures and images for: Identification of Candidate Adaxial–Abaxial-Related Genes Regulating Petal Expansion During Flower Opening in Rosa chinensis “Old Blush”
Source: Front Plant Sci. 2019 Sep 10;10:1098. doi: 10.3389/fpls.2019.01098 (PMC6747050; doi:10.3389/fpls.2019.01098)

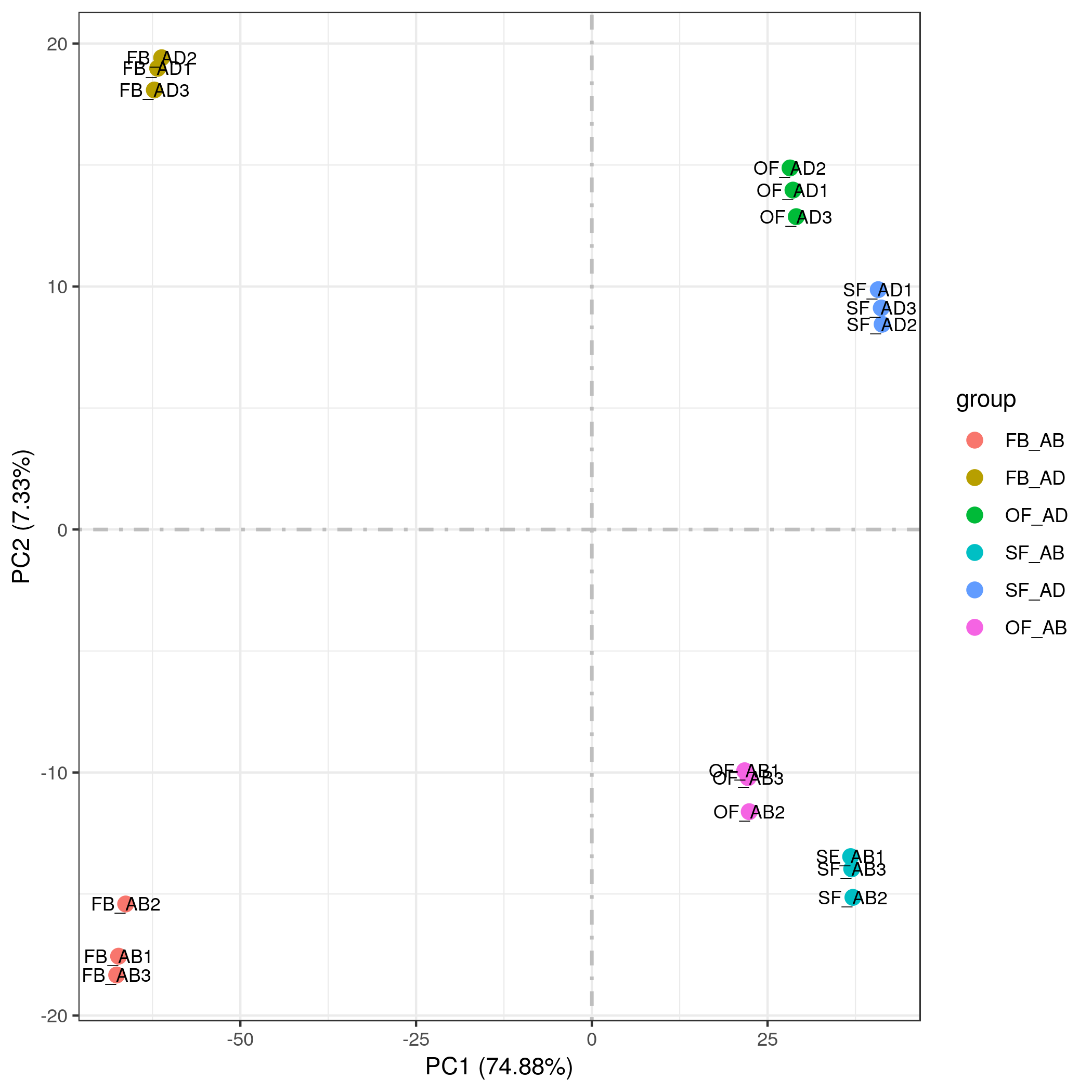

Supplement: Supplementary file 1 [file DataSheet_1.zip › Supplementary Information/Supplemental Figure 1.tif]

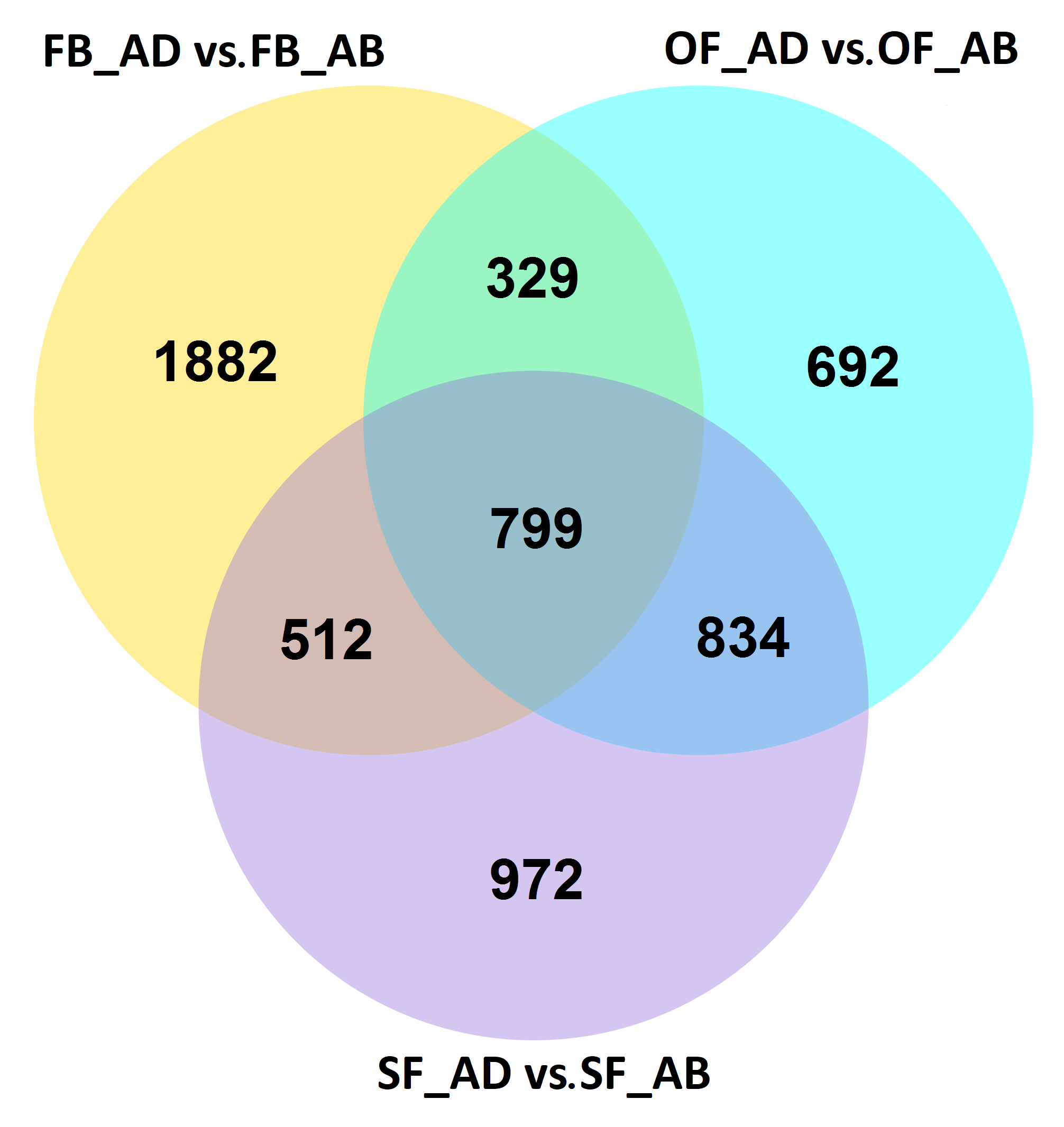

Supplement: Supplementary file 1 [file DataSheet_1.zip › Supplementary Information/Supplemental Figure 2.tif]

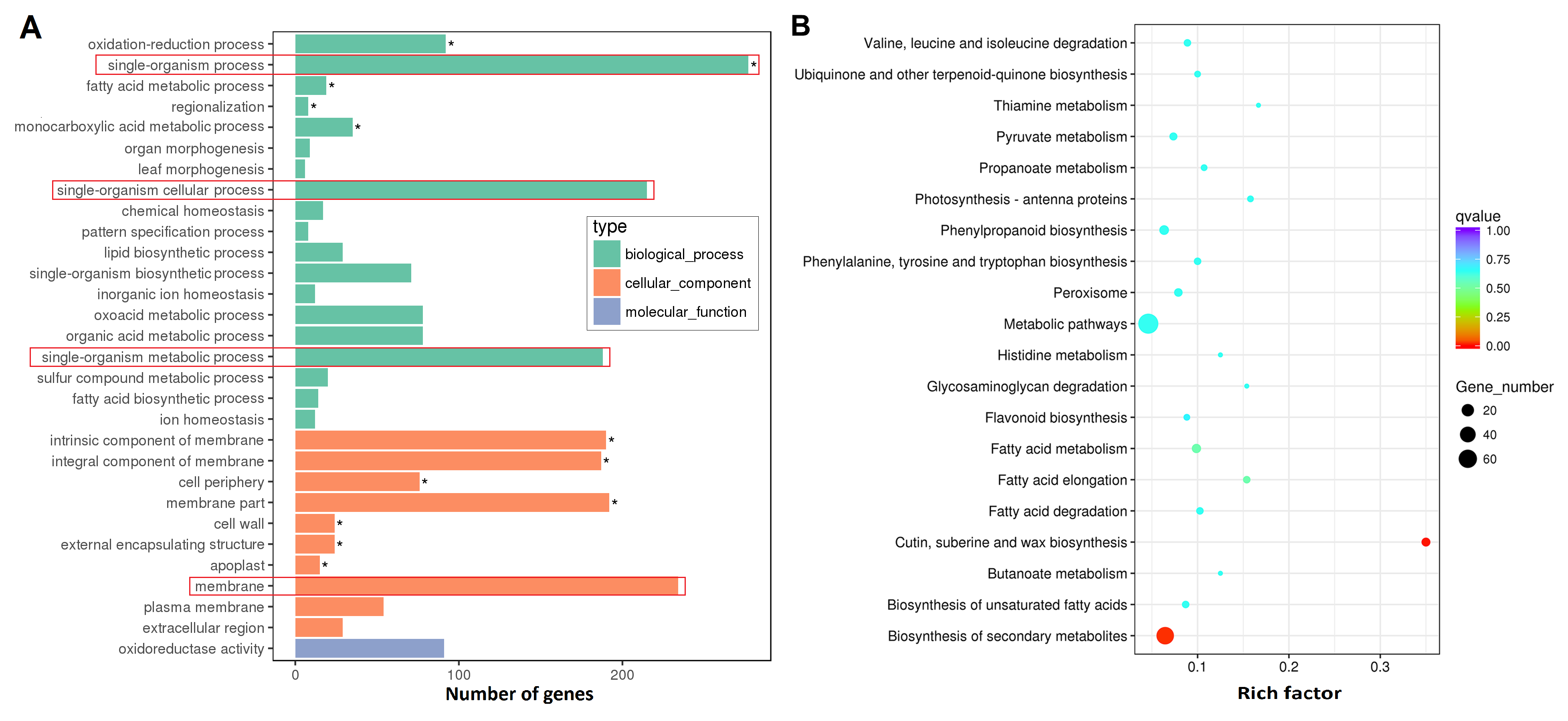

Supplement: Supplementary file 1 [file DataSheet_1.zip › Supplementary Information/Supplemental Figure 3.tif]

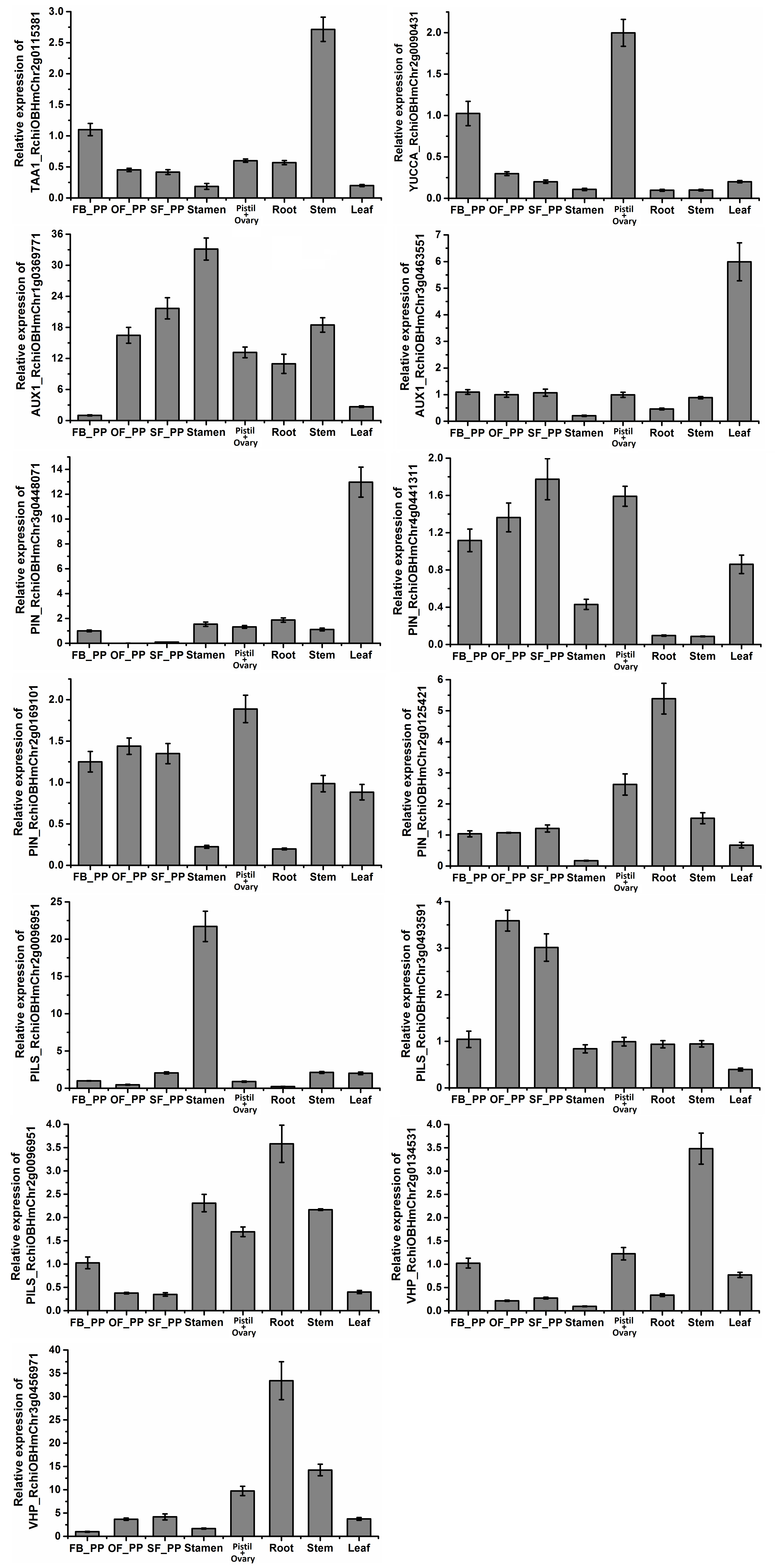

Supplement: Supplementary file 1 [file DataSheet_1.zip › Supplementary Information/Supplemental Figure 4.tif]

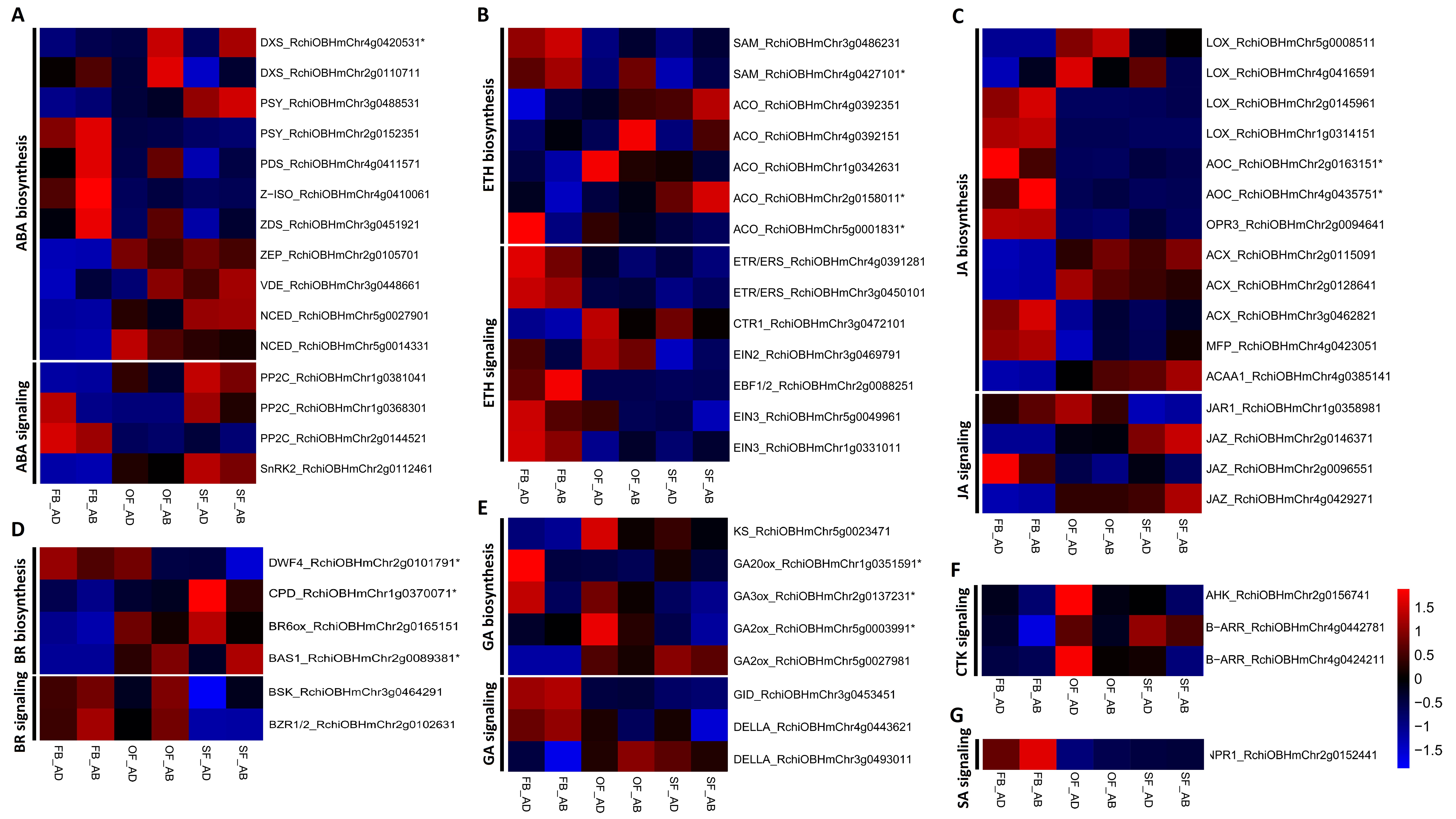

Supplement: Supplementary file 1 [file DataSheet_1.zip › Supplementary Information/Supplemental Figure 5.tif]

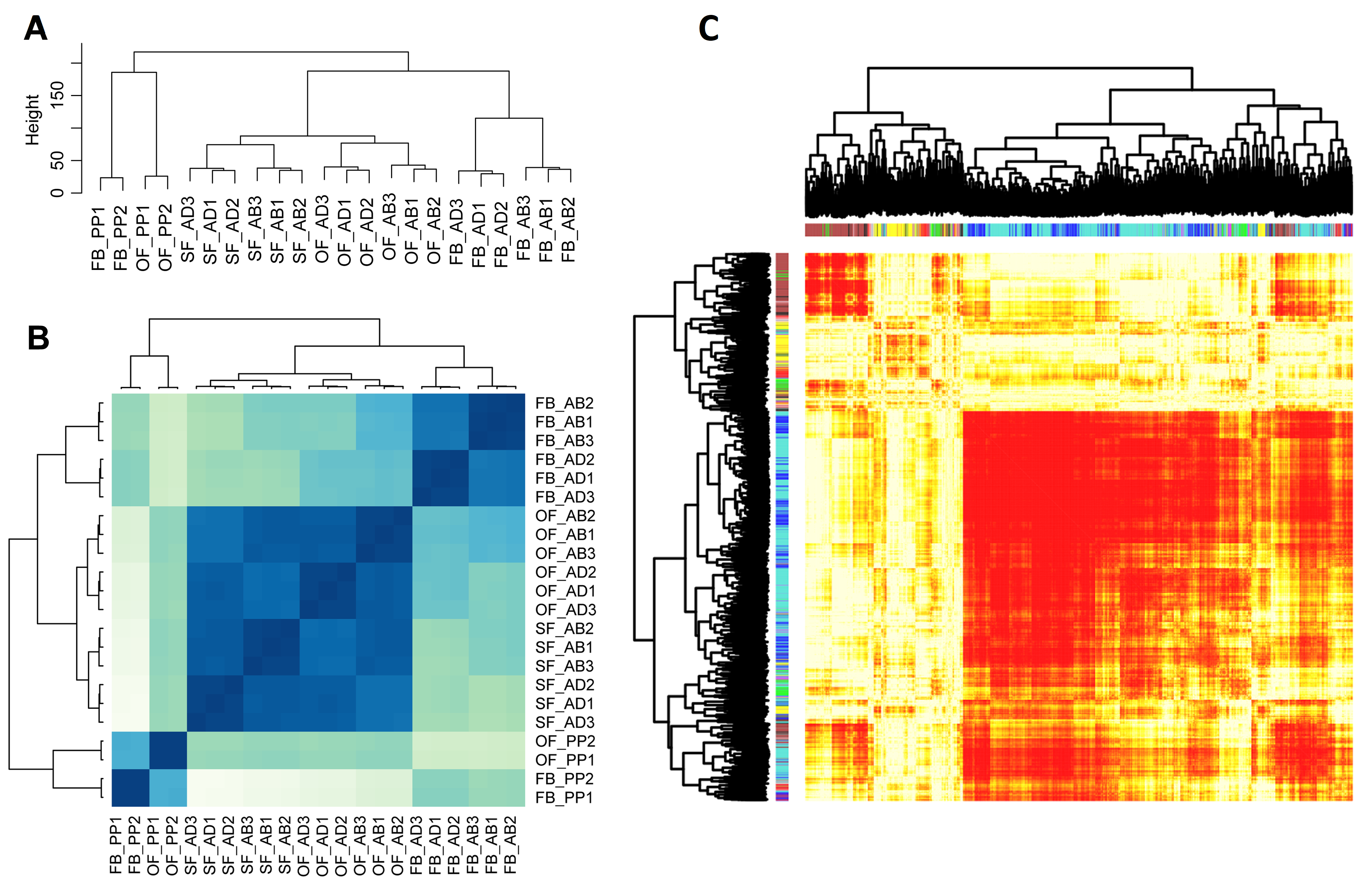

Supplement: Supplementary file 1 [file DataSheet_1.zip › Supplementary Information/Supplemental Figure 6.tif]

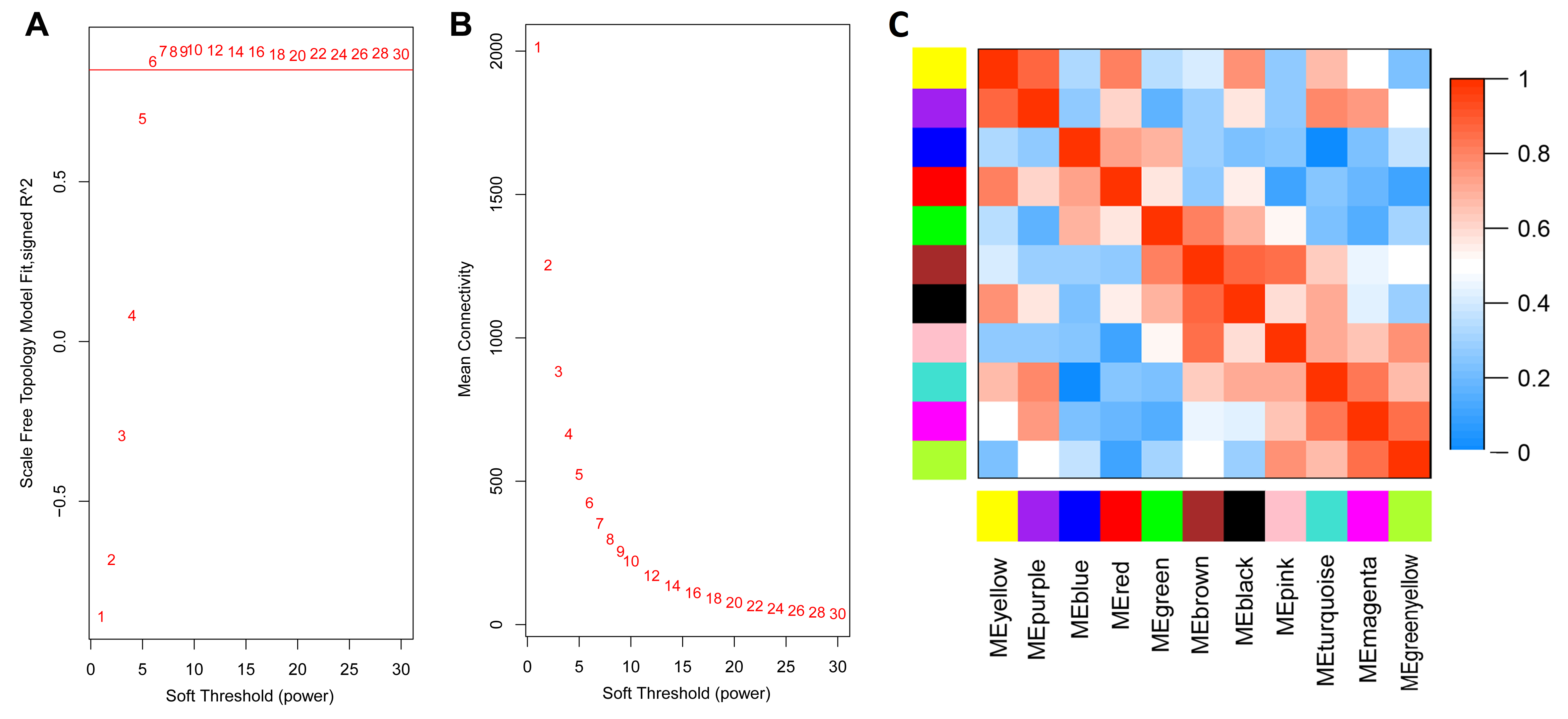

Supplement: Supplementary file 1 [file DataSheet_1.zip › Supplementary Information/Supplemental Figure 7.tif]

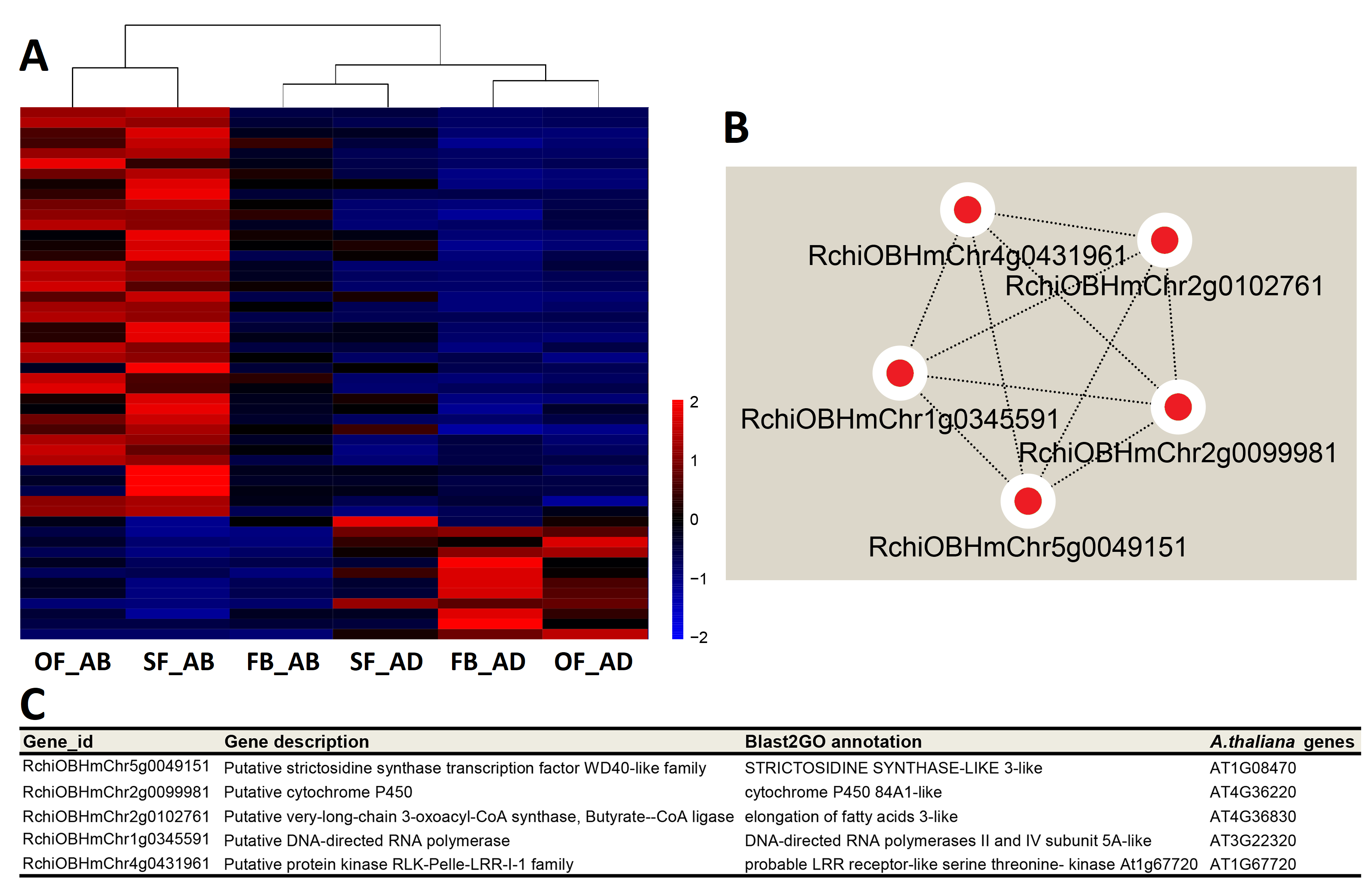

Supplement: Supplementary file 1 [file DataSheet_1.zip › Supplementary Information/Supplemental Figure 8.tif]
